# Imaging of Inflammation in Spinal Cord Injury: Novel Insights on the Usage of PFC-Based Contrast Agents

**DOI:** 10.3390/biomedicines9040379

**Published:** 2021-04-03

**Authors:** Francesca Garello, Marina Boido, Martina Miglietti, Valeria Bitonto, Marco Zenzola, Miriam Filippi, Francesca Arena, Lorena Consolino, Matilde Ghibaudi, Enzo Terreno

**Affiliations:** 1 Molecular and Preclinical Imaging Centers, Department of Molecular Biotechnology and Health Sciences, University of Torino, 10126 Torino, Italy; francesca.garello@unito.it (F.G.); valeria.bitonto@unito.it (V.B.); marcozenzola@gmail.com (M.Z.); filippimiriam@hotmail.com (M.F.); Francesca.Arena@bracco.com (F.A.); lconsolino@ukaachen.de (L.C.); 2 Department of Neuroscience “Rita Levi Montalcini”, University of Turin, 10126 Turin, Italy; matilde.ghibaudi@unito.it; 3 Neuroscience Institute Cavalieri Ottolenghi, University of Turin, 10043 Orbassano (TO), Italy; martina.miglietti@edu.unito.it; 4 National Institute of Neuroscience (INN), 10126 Turin, Italy; 5 Institute of Biostructures and Bioimaging (IBB), Italian National Research Council (CNR), 10126 Torino, Italy

**Keywords:** M1-M2 macrophages, spinal cord injury, 19F magnetic resonance imaging, macrophage tracking, macrophage polarization, PFCE, Kupffer cells saturation, PRESS, nanoemulsion, inflammation imaging

## Abstract

Labeling of macrophages with perfluorocarbon (PFC)-based compounds allows the visualization of inflammatory processes by ^19^F-magnetic resonance imaging (^19^F-MRI), due to the absence of endogenous background. Even if PFC-labeling of monocytes/macrophages has been largely investigated and used, information is lacking about the impact of these agents over the polarization towards one of their cell subsets and on the best way to image them. In the present work, a PFC-based nanoemulsion was developed to monitor the course of inflammation in a model of spinal cord injury (SCI), a pathology in which the understanding of immunological events is of utmost importance to select the optimal therapeutic strategies. The effects of PFC over macrophage polarization were studied in vitro, on cultured macrophages, and in vivo, in a mouse SCI model, by testing and comparing various cell tracking protocols, including single and multiple administrations, the use of MRI or Point Resolved Spectroscopy (PRESS), and application of pre-saturation of Kupffer cells. The blood half-life of nanoemulsion was also investigated by ^19^F Magnetic Resonance Spectroscopy (MRS). In vitro and in vivo results indicate the occurrence of a switch towards the M2 (anti-inflammatory) phenotype, suggesting a possible theranostic function of these nanoparticles. The comparative work presented here allows the reader to select the most appropriate protocol according to the research objectives (quantitative data acquisition, visual monitoring of macrophage recruitment, theranostic purpose, rapid MRI acquisition, etc.). Finally, the method developed here to determine the blood half-life of the PFC nanoemulsion can be extended to other fluorinated compounds.

## 1. Introduction

Spinal cord injury (SCI) is a traumatic event characterized by an acute central nervous system (CNS) physical injury followed by tissue damage occurring at various extents in the following days and months, resulting in permanent loss of sensory and motor function below the injury level [1]. Extensive literature describes the crucial involvement of both CNS resident immune cells (microglia) and recruited macrophages in the perpetuation of this damage [2,3,4]. However, the contribution of these cells could be not only detrimental, but also beneficial, as they have the potential of promoting tissue remodeling and axon regeneration [5]. Microglia and macrophages, in fact, can be polarized either towards a pro-inflammatory M1 or a pro-regenerative M2 phenotype, depending on the surrounding microenvironment [6]. Since at present the only clinical treatments available for SCI, consisting of stabilization and decompression of spinal cord combined with a high dose of methylprednisolone, are still debated due to the limited beneficial effects, research for innovative therapies is still ongoing [7]. Besides implantable brain–computer interface technologies [8], cell therapy is one of the most promising approaches as the graft of stem cells has already provided valuable pre-clinical data about their regenerative potential in SCI animal models [4,9,10]. However, since the acute inflammatory process following the traumatic event is not favorable for survival and differentiation of transplanted cells, as well as the glial scar formation occurring at a later stage (subacute/intermediate phase) inhibits axonal regeneration, the successful outcome of cell therapies is strictly dependent on the timing of their administration [11]. Methods for non-invasive inflammation monitoring are of paramount importance to define the optimal therapeutic window. Among the various imaging techniques available, ^19^F Magnetic Resonance Imaging (MRI) offers many advantages. The excellent spatial resolution of MRI associated with the high avidity of immune system cells towards certain fluorinated agents and the absence of endogenous fluorine signal are three fundamental aspects [12]. Nanosystems based on perfluorocarbon (PFC) have been extensively used to track immune cells in various inflammatory processes [13,14]. ^19^F MRI has been applied in both clinical [15] and pre-clinical studies to monitor cardiac and cerebral ischemia [16], diabetes [17], inflammatory bowel disease (IBD) [18], arthritis [19,20], experimental allergic encephalomyelitis (EAE) [21,22], and other inflammatory conditions. Ex-vivo labeling of T cells or dendritic cells (DC) and in vivo labeling of monocytes/macrophages are the most commonly applied approaches. In vivo labeling of cells, following systemic injection of fluorinated systems, is generally achieved by nanoparticles being taken up by the phagocytic cells of the reticuloendothelial system (RES), including circulating blood monocytes and tissue macrophages (and in much smaller numbers, neutrophils, and DCs), which are attracted to inflammatory injury sites [23]. Phenotype variations have never been reported in DCs and T cells labeled with fluorinated nanoparticles [24,25] with size below 500 nm [26], whereas the impact of PFC on macrophage polarization has never been investigated, neither in vitro nor in vivo. In the present study, a perfluoro-15-crown-5 ether (PFCE)-based nanoemulsion (PFCE-NE) was used to label and track immune system cells in a murine model of SCI to monitor the course of inflammation in vivo. The ability of this fluorinated emulsion to polarize macrophages towards the anti-inflammatory (M2) phenotype, both in vitro and in vivo, was evaluated. Besides, the blood half-life of this system was investigated for the first time, through a ^19^F Magnetic Resonance Spectroscopy (MRS) method allowing for non-invasive estimation of blood clearance of fluorinated compounds. Moreover, as a huge amount of the administered PFCE-NE is generally sequestered by Kupffer cells in the liver, pre-saturation of these cells using phospholipid-based vesicles was applied to increase the bioavailability of the ^19^F-based agent at the lesion site. Finally, imaging studies were carried out to test and compare different protocols of PFCE-NE administrations. Thus, our research brings new insights in imaging inflammation via ^19^F-MR, shedding light on various aspects never addressed before.

## 2. Experimental Section

### 2.1. Animal Care and Use

C57BL/6J male mice (8–12 weeks old) were obtained from the animal facility of the Molecular Biotechnology Center (MBC) of the University of Turin (Italy) and used to obtain macrophages for in vitro experiments, as well as to generate the SCI models. Experiments were performed according to the national laws on animal experimentation and approved by the Italian Ministry of Health (Direzione Generale della sanità animale e dei farmaci veterinari) (project identification: 1109/2015-PR, date of approval: 19/10/2015). Mice were kept in standard housing with standard rodent chow and water available ad libitum, and a 12 h light/dark cycle. In order to perform surgery and imaging, mice were anesthetized by intramuscular injection of a combination of 20 mg/kg tiletamine/zolazepam (Zoletil 100; Virbac, Milan, Italy) and 5 mg/kg xylazine (Rompun; Bayer, Milan, Italy).

### 2.2. PFCE-NE Synthesis and Characterization

The PFCE-NE was obtained by mixing 63 µL/mL of Perfluoro-15-crown-5 ether (Exfluor Research Corporation, Round Rock, TX, USA) with 0.45 mg/mL 1,2-dioleoyl-sn-glycero-3-phosphoethanolamine-*N*-(lissamine rhodamine B sulfonyl) (ammonium salt) (Rhodamine-DOPE, Avanti Polar Lipids Inc., Birmingham, AL, USA), and Kolliphor^®^ P188 (Merck KGaA, Darmstadt, Germany) (10% *w/v* in HEPES buffer (NaCl 0.15 M, HEPES 3.8 mM, pH 7.2–7.4)). To obtain the emulsion, sonication of the suspension was performed with an electronic sonopuls UW2070 sonicator tip (BANDELIN electronic GmbH & Co. KG, Berlin, Germany) six times for 1 min at 75% power (52.5 Watt), in a cold-water bath. At the end of the sonication process, the mean size of the particles was determined by dynamic light scattering (DLS) using a Malvern Zetasizer 3000HS (Malvern, UK) (sample dilution 1:100 in HEPES buffer), then the pH was measured and adjusted to 7.2 ± 0.2. Chemical shift with respect to trifluoroacetic acid (TFA), longitudinal (T_1_), and transversal (T_2_) relaxation times were determined by ^19^F Nuclear Magnetic Resonance (NMR) on a 1:50 solution of PFCE-NE in HEPES buffer at 7 T, 25 °C. As the stability of the preparation at 4 °C was limited due to the naturally occurring aggregation process, PFCE-NE was sonicated three times for 1 min at 75% power, and size was determined by DLS immediately before use.

### 2.3. Liposome Synthesis and Characterization

In order to pre-saturate liver Kupffer cells, liposomes formulated as follows: DPPC (1.2-Dipalmitoyl-sn-glycero-3-phosphocoline, Avanti Polar Lipids Inc., Birmingham, AL, USA)/DSPE-PEG2000 (1.2 Distearoyl-sn-glycero-3-phosphoethanolamine-*N*-(methoxy(polyethyleneglycol)-2000) ammonium salt, Avanti Polar Lipids Inc., Birmingham, AL, USA), in ratio 95:5, were prepared via the film hydration method. Briefly, phospholipids were dissolved in chloroform, and then the solvent was evaporated under vacuum through a Rotavapor, so as a thin film formed in the round bottom flask. The film was then hydrated at 55 °C with 1 mL of HEPES buffer (to obtain a final phospholipid concentration of 40 mg/mL) and the resulting colloidal suspension was extruded through polycarbonate filters of decreasing pore diameters (from 800 to 200 nm, LIPEX extruder, Northern Lipids, Inc., Burnaby, BC, Canada). The size and polydispersity index (PDI) of liposomes were determined by DLS. Liposomes were dialyzed overnight against isotonic HEPES buffer.

### 2.4. Cell Extraction and Polarization

For murine bone marrow-derived macrophage isolation, mice were anesthetized and killed by cervical dislocation. Immediately after death, femurs and tibias were excised, cleaned from the connective tissue and muscles, and the bone epiphyses were cut away. Bone cavities were flushed with 2 to 5 mL of DMEM/F-12 (Dulbecco’s Modified Eagle Medium/Nutrient Mixture F-12, EuroClone, Milano, Italy) medium, until appearing white. Clumps of cells and tissue debris were removed by filtering the suspension through a 70 µm cell strainer (BD Biosciences Pharmingen, San Diego, CA, USA). The filtered suspension was centrifuged (10 min, 500× *g*), the supernatant discarded and the pellet of bone marrow-derived cells was re-suspended in 4 mL of DMEM/F-12 supplemented with 10% (*v*/*v*) heat-inactivated fetal bovine serum (FBS), 12.5 mM L-glutamine, 100 U/mL penicillin and 100 μg/mL streptomycin. Bone marrow-derived cells were seeded on sterile pre-treated glass coverslips placed into 24 wells cell culture plates at a density of around 3 × 10^5^ cells/well. Cells were incubated for 7 days at 37 °C, 5% CO_2_, with 20 ng/mL of macrophage colony-stimulating factor (M-CSF; Merck KGaA, Darmstadt, Germany) to obtain adherent non-polarized-M0 macrophages. After 7 days, non-adherent cells were removed by extensive washing with Phosphate Buffered Saline (PBS) and macrophage polarization was induced by incubating adherent M0 cells for 48 h at 37 °C in DMEM/F-12 culture medium supplemented with 10 mM L-glutamine, 10% FBS, 100 U/mL penicillin, and 100 μg/mL streptomycin. In order to obtain M1-polarized cells, the medium was supplemented with 100 ng/mL LPS (Lipopolysaccharide, Merck KGaA, Darmstadt, Germany) and 20 ng/mL IFNγ (Interferon gamma, PeproTech, London, UK), while to obtain M2-polarized macrophages medium was supplemented with 20 ng/mL of IL-4 (Interleukin 4, PeproTech, London, UK), and 20 ng/mL IL-10 (Interleukin 10, PeproTech, London, UK).

### 2.5. PFCE-NE In Vitro Experiments

Macrophages before (M0 macrophages) or at the end of the polarization process (M1 and M2 macrophages) were washed and incubated at 37 °C with the PFCE-NE suspension, at a concentration of 2 mM (40 mM ^19^F) [26] in DMEM/F-12 supplemented with 10 mM L-glutamine, 100 U/mL penicillin, and 100 μg/mL streptomycin for 1 h, 6 h, and 24 h. At the end of the incubation time, cells were washed five times with PBS, fixed with 4% paraformaldehyde (PFA) (15 min, Room Temperature, RT), washed two times with PBS, and stored in the dark at 4 °C. Non-incubated cells (M0, M1 and M2) were used as control and fixed as above-mentioned. Images of cell morphology were taken with a Zeiss Axio Observer microscope (Carl Zeiss AG, Oberkochen, Germany). 

^1^H/^19^F MRI was performed on M0, M1, and M2 cells incubated at 37 °C with the PFCE-NE suspension (2 mM) for 1 h. To this purpose, at the end of the incubation cells were washed five times with PBS, detached with Cell Dissociation Solution Non-enzymatic 1× (Sigma Aldrich), and centrifuged (1100 rpm, 5 min, 4 °C). Further, the cells were resuspended in 50 µL of PBS, transferred into glass capillaries, and centrifuged at 700× *g* rpm for 10 min to obtain a cell pellet. The capillaries were then inserted into an agar phantom and imaged at 7.1 T with a Bruker Avance 300 spectrometer equipped with a dual ^1^H/^19^F imaging probe using the Bruker Paravision 5.1 software (Billerica, MA, USA). For ^1^H MRI fast T_2_ weighted (T_2w_) coronal images were acquired with the following parameters: Echo Time (TE) = 3.49 ms, Repetition Time (TR) = 4000 ms, Number of Averages (NAV) = 2, Rare Factor (RF) = 32, matrix Size = 128 × 128, Field of View (FOV) = 3.00 × 3.00 cm, slice thickness = 3.00 cm, acquisition time = 32 s. For ^19^F MRI ^19^F basic frequency (SFO1), and relative P1 values were calculated and the following sequence was used: TE = 2.96 ms, TR = 1000 ms, NAV = 4000, RF = 24, matrix Size = 32 × 32, FOV = 3.00 × 3.00 cm, slice thickness = 3.00 mm, acquisition time = 1 h 6 min.

For immunofluorescence, cell-covered glass coverslips were moved gently with tweezers, lifted, and placed on parafilm in the humidified chamber. Then cells were washed three times with PBS and unspecific binding site blocking was carried out with 5% Normal Donkey Serum (NDS) for 30 min at RT. Incubation with primary antibodies was performed (NDS 2%; CD206 goat anti-mouse 1:200 R&D Systems, AF2535; CD86 rat anti-mouse 1:100 BD Pharmingen, 553689), overnight, at 4 °C. The day after, cells were washed three times with PBS and incubated for 1 h at RT with secondary antibodies: 488 Donkey anti-Rat 1:200 (Jackson ImmunoResearch) and 647 Donkey anti-goat 1:200 (Jackson ImmunoResearch) or PBS. Cells were then washed three times with PBS, then nuclei were stained with 4′,6-diamidino-2-phenylindole (DAPI; 1:1000 in PBS 1×) for 3 min at RT. Finally, cells were washed with PBS and mounted on bigger glass coverslips with ProLong™ Gold Antifade Mountant (Thermo Fisher Scientific, Milano, Italy). Confocal microscopy analysis was performed after one day using Laser Scanning Confocal Microscopy (Leica Microsystem Srl., Milano, Italy), objective 40× with oil immersion.

To quantify the number of CD206 and/or CD86 positive cells, three random images were acquired for each sample (*n* = 3). Cells incubated with secondary antibody only were used as control, to select a signal threshold. The number of nuclei and that of antigen-positive cells was counted using ImageJ 1.52i (National Institutes of Health, Bethesda, MD, USA). Afterward, the percentage of PFCE-NE^+^/PFCE-NE^-^ cells was calculated over the total number of cells, using the formula below:

(1) $${\%\;\mathrm{PFCE}-\mathrm{NE}}^{+}\;\mathrm{cells}\;=\left(\frac{{\mathrm{number}\;\mathrm{of}\;\mathrm{PFCE}-\mathrm{NE}}^{+}\mathrm{cells}\;}{\mathrm{total}\;\mathrm{number}\;\mathrm{of}\;\mathrm{cells}}\right)\times 100$$

Besides, the percentage of CD206^+^, CD86^+^, CD86^+^/CD206^+^, CD86^−^/CD206^−^ cells was calculated over the number of PFCE-NE^+^ cells. To calculate these values the following formula was used:

(2) $${\%\;\mathrm{PFCE}-\mathrm{NE}}^{+}/{\mathrm{CDn}}^{+/-}\;\mathrm{cells}\;=\left(\frac{{\mathrm{number}\;\mathrm{of}\;\mathrm{CDn}}^{+/-}\mathrm{cells}\;}{{\mathrm{number}\;\mathrm{of}\;\mathrm{PFCE}-\mathrm{NE}}^{+}\;\mathrm{cells}}\right)\times 100$$

where *n* stands for 86, 206, or 86/206. In both cases mean values of the three acquisitions were then calculated.

### 2.6. Blood Half-Life Time Determination by ^19^F MRS

C57BL/6J healthy mice (*n* = 6) were anesthetized and a catheter was inserted into the tail vein. The mice were positioned into a Bruker Avance 7 T MRI scanner (Bruker BioSpin MRI Ettlingen, Germany) operating at frequencies of 300 MHz for ^1^H and 282.38 MHz for ^19^F measurements. Allocation of animals in the scanner was performed accurately to ensure that positioning and imaging could be consistent through all imaging sessions. Only the head of the animal was included in the volume coil. The respiratory rate was continuously monitored using a respiratory air pillow (SA Instruments, Stony Brook, NY, USA). PFCE-NE was injected via the tail vein (1 mmol/kg body weight (b.w.)). Immediately after the injection, ^19^F NMR spectra were acquired every 43 s for 1 h. Additional spectra were acquired 3, 24, 48, and 72 h after the injection. A standard reference tube containing TFA (65.3 mM) was used to normalize the signal acquired during each imaging session. Acquisition parameters were: Relaxation delay (D1) = 2.0 s; Number of Scans (NS) = 8; Acquisition Time = 3.30 s; P1 (Pulse) = 60 µs; PL1 (Power Level) = −4.0 dB; Receiver gain = 1150; Sweep Width (SW) = 19,841 Hz. The area under the curve (AUC) was then calculated in each spectrum (using TFA as standard) and plotted with time. Fitting of acquired data was carried out to estimate the blood half-life time of the system. The procedure was repeated exactly in the same way in the case of pre-saturation of Kupffer cells (see paragraph 2.9), with the addition of intravenous (i.v.) liposome injection (same volume as PFCE-NE) 10 min before PFCE-NE. The absence of PFCE-NE signal in the brain was demonstrated by confocal microscopy on ex-vivo brain tissue slices collected 24 h post PFCE-NE injection (see Section 2.10 for tissue processing details).

### 2.7. ^19^F/^1^H MRI of Blood Samples

In order to label monocytes/macrophages with PFCE-NE in vivo, the suspension was i.v. administered to healthy mice (*n* = 2, b.w. 20 g), 1 mmol/kg. Three hours post-injection the mice were anesthetized, blood was collected from the vena cava inferior and transferred into heparin-loaded cones. Blood was then diluted 1:1 in PBS, transferred into a 15-mL falcon, and separated using the Ficoll–Histopaque methodology. Briefly, blood was stratified into Ficoll–Histopaque and centrifuged for 25 min at 1500× *g* rpm and 25 °C. At the end of the centrifugation process, the falcon was imaged by ^1^H/^19^F MRI. For ^1^H MRI fast T_2_ weighted (T_2w_) coronal images were acquired with the following parameters: Echo Time (TE) = 3.49 ms, Repetition Time (TR) = 4000 ms, Number of Averages (NAV) = 2, Rare Factor (RF) = 32, matrix Size = 128 × 128, Field of View (FOV) = 3.00 × 3.00 cm, slice thickness = 3.00 cm, acquisition time = 32 s. For ^19^F MRI ^19^F basic frequency (SFO1), and relative P1 values were calculated and the following sequence was used: TE = 5.64 ms, TR = 1500 ms, NAV = 384, RF = 32, matrix Size = 32 × 32, FOV = 3.00 × 3.00 cm, slice thickness = 3.00 mm, acquisition time = 9 min 36 s.

### 2.8. SCI Model

To perform SCI, C57BL/6J male mice were anesthetized and placed under an optical microscope. Then, the lower thoracic and lumbar spine was exposed and the spinal muscles displaced laterally. A complete spinal cord (SC) transection was performed at T13 level, by using a 27 1/2-gauge needle (SCI mice, *n* = 28). Then, the animals were sutured and the wound was disinfected. A group of healthy (no surgery was performed) mice was used as control (*n* = 6) (see paragraph 2.9).

### 2.9. Administration of PFCE to SCI Mice and In Vivo ^19^F MRI

**Figure 1:**
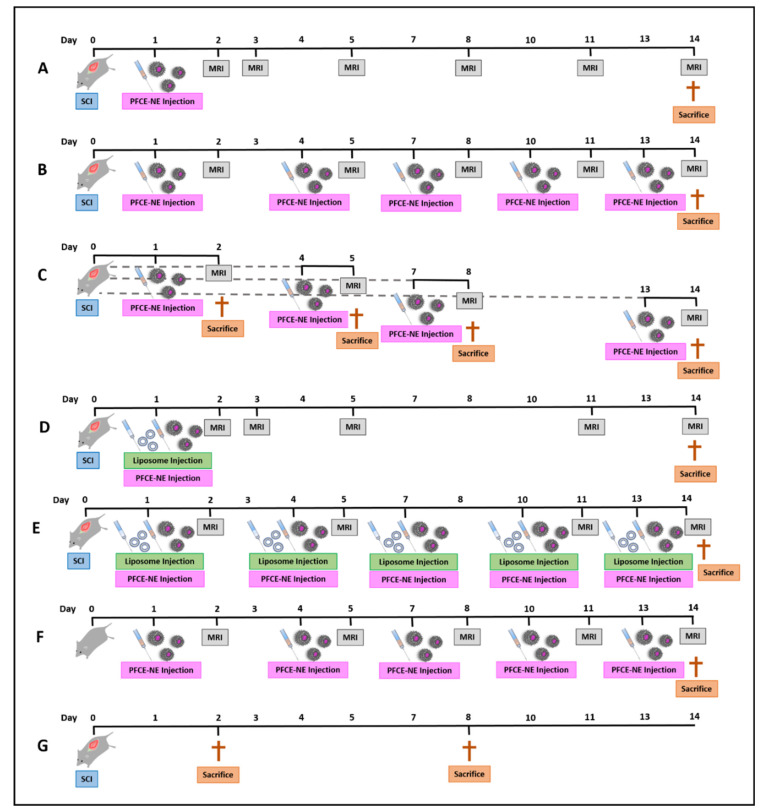
Schematic representation of the different experimental groups: (**A**) SCI mice, single PFCE-NE administration at 1 DPI, MRI at 2, 3, 5, 8, 11, and 14 DPI; (**B**) SCI mice, multiple PFCE-NE administrations at 1, 4, 7, 10, 13 DPI, MRI at 2, 5, 8, 11, and 14 DPI; (**C**) SCI mice, single PFCE-NE administration at 1, 4, 7, or 13 DPI, MRI at 2, 5, 8, or 14 DPI; (**D**) SCI mice, single PFCE-NE administration at 1 DPI with Saturation of Kupffer Cells, MRI at 2, 3, 5, 11, and 14 DPI; (**E**) SCI mice, multiple PFCE-NE administrations at 1, 4, 7, 10, 13 DPI, with Saturation of Kupffer Cells, MRI at 2, 5, 11, and 14 DPI; (**F**) Healthy Mice, multiple PFCE-NE administrations at 1, 4, 7, 10, 13 DPI, MRI at 2, 5, 8, 11, and 14 DPI; (**G**) SCI mice, no PFCE-NE administration, no MRI.

SCI and healthy mice were divided into seven different groups (Figure 1). Except for Group 7, all the other groups received PFCE-NE by i.v. injection at a dosage of 1 mmol/Kg b.w. (20 mmol ^19^F/Kg b.w.) and were imaged by ^1^H/^19^F MRI starting from 24 h post-injection. Group 1: (*n* = 3): SCI mice received a single dose of PFCE-NE at day 1 post-injury (p.i.), were imaged at 2, 3, 5, 8, and 14 days post-injury (DPI), and sacrificed at 14 DPI (Figure 1A). Group 2: SCI mice (*n* = 3) received multiple administrations of PFCE-NE (1, 4, 7, 10, 13 DPI), were imaged at 2, 5, 8, 11, and 14 DPI, and sacrificed at 14 DPI (Figure 1B). Group 3: SCI mice (*n* = 12) received a single dose of PFCE-NE at 1, 4, 7, or 13 DPI, were imaged the day after the injection, and sacrificed immediately after (Figure 1C). Group 4 and 5: SCI mice (*n* = 6) were treated with the same protocol as group 1 and 2, respectively, but with the addition of pre-saturation of Kupffer cells through the i.v. injection of liposomes (same volume as PFCE-NE) 10 min before PFCE-NE administration (Figure 1D,E). At 8 DPI, no MRI acquisition was performed due to technical problems. Group 6: healthy mice (*n* = 3) received multiple administrations of PFCE-NE (day 1, 4, 7, 10, 13 post-enrollment), were imaged at day 2, 5, 8, 11, and 14 post-enrollment, and sacrificed at the 14th day (Figure 1F). Group 7: SCI mice (*n* = 4) did not receive PFCE-NE and consequently were not imaged by ^19^F MRI due to the absence of signal background, but were sacrificed at 2 or 8 DPI for ex-vivo studies (Figure 1G).

For ^1^H MRI T_2w_ axial images were acquired with the following parameters: TE = 4.85 ms, TR = 4000 ms, NAV = 2, RF = 32, matrix Size = 256 × 256, FOV = 3.50 × 3.50 cm, slice thickness = 3.00 cm, number of slices = 3, acquisition time = 1 min 4 s. For ^19^F MRI a RARE (Rapid Acquisition with Relaxation Enhancement) sequence was used: TE = 2.98 ms, TR = 1000 ms, NAV = 3600, RF = 24, matrix Size = 32 × 32, FOV = 3.50 × 3.50 cm, slice thickness = 3.00 mm, number of slices = 3, acquisition time = 1 h. For ^19^F MRI ^19^F basic frequency (SFO1) and relative P1 values were calculated before every imaging session. Selected regions of interest (ROIs) were manually drawn using ParaVision 5.1 on the spinal cord and the amount of fluorine in each ROI was calculated using a standard reference tube placed next to the mouse (2.02 mM PFCE-NE in agar). The volume of the reference tube acquired in a single slice was 33 mm^3^, corresponding to 66.7 nmol of PFCE-NE, so as the moles of ^19^F in the lesion site were then calculated in each slice as follows:

(3) nmol F 19= SISCISIref × 66.7 × 20

where SI stands for Signal Intensity, 66.7 corresponds to nmol of PFCE-NE in the reference tube imaged in each slice and 20 to the number of fluorine atoms in PFCE.

In Group 2, the % Fluorine Enhancement has been calculated as follows:

(4) % Fluorine Enhancement= nmol F 19day n−nmol F 19day of previous MRInmol F 19day of previous MRI × 100

where *n* is the day of the last MRI acquisition, and with the enhancement set as 100% for day 2 (no previous MRI acquisition available and no PFCE-NE previously injected). 

For ^1^H and ^19^F images superimposition, the matrix of ^19^F images was resized post-acquisition to 128 × 128 or 256 × 256, the hot iron scale was selected and then the overlay on ^1^H greyscale images was carried out using ParaVision 5.03, ImageJ, or AMIDE 1.0.4.

To perform Point Resolved Spectroscopy (PRESS), ^1^H localized shim was optimized on a 3.50 mm × 3.50 mm × 3.50 mm (42.87 mm^3^) voxel centered on the lesion. Then ^19^F PRESS was carried out on the shimmed voxel with the following parameters: TE = 13.64 ms, TR = 4000 ms, NS = 270, Spectral width = 280 ppm, number of points = 2048, Acquisition Time = 18 min 16 s. The same procedure was applied on a 2.50 mm × 2.50 mm × 2.50 mm (15.62 mm^3^) voxel centered on the reference tube containing 2.02 mM PFCE-NE in agar. 

### 2.10. Tissue Processing

To perform histological analysis, animals were deeply anesthetized and transcardially perfused with 0.1 M Phosphate Buffer (PB), pH 7.4, followed by 4% buffered paraformaldehyde (PFA, pH 7.4) in the same PB. Brain and spinal cord were collected. The spinal cord was then cut between T11 and L2 vertebral segments and removed. The spinal cord was post-fixed in PFA for 2 h at 4 °C. Samples were then transferred overnight in 30% sucrose in phosphate buffer 0.1 M at 4 °C for cryoprotection. Then, they were embedded in optimal cutting temperature (OCT) compound (Killik; Bio-Optica, Milan, Italy) at −20 °C and cut at the cryostat. Moreover, 50 μm-thick sections were then cut longitudinally. Finally, the sections were transferred into 12-well plates and maintained in a cryoprotective glycerol-based solution. The plates were stored at −20 °C.

### 2.11. Immunofluorescence Reactions on Spinal Cord Sections

Sections were washed 3 times for 5 min with PBS 1× on a tilting shaker and then immersed in PBS-Triton 0.3% for permeabilization. After 20 min, sections were washed in PBS. Afterward, a blocking solution was used (90% PBS Triton 0.3%; 10% NDS, Sigma-Aldrich, Milan, Italy) to block unspecific binding sites, for 30 min, at RT on a tilting shaker. Then, the incubation with the primary antibodies was performed (PBS Triton 0.3%; NDS 10%; goat anti-mouse CD206, 1:200, R&D Systems, AF2535; rat anti-mouse CD86, 1:100, BD Pharmingen, 553689; goat anti-mouse IBA1, 1:1000, Wako, 011-27991) overnight, at 4 °C, on the tilting shaker. The following day, 3 washes in PBS 1× were performed, followed by incubation of secondary antibodies diluted in PBS 1× and NDS 2% (647 donkey anti-goat 1:200; 488 donkey anti-rat 1:200; Jackson ImmunoResearch) for 90 min on a tilting shaker, at RT. Afterward, 3 washes in PBS 1× were performed, followed by incubation with DAPI (1:1000 in PBS 1×) for 3 min, at RT, on a tilting shaker, to label nuclei. Sections were washed five times in PBS 1×, gently transferred on slides, and coverslipped with the anti-fade mounting medium Mowiol. Confocal microscopy analysis was then performed using Laser Scanning Confocal Microscopy (Leica TCS SP5). Fluorescence microscopy images were acquired with a Nikon Eclipse 80i microscope.

Three random images were acquired in the lesion area of the spinal cord for each animal; PFCE-NE+ and antigen-positive cells were counted and the number of cells per field of view reported. Percentages of CD86^+^/PFCE-NE^+^, CD206^+^/PFCE-NE^+^, CD86^+^/PFCE-NE^-^, CD206^+^/PFCE-NE^-^ cells were then calculated over the total cell number of cells positive to CD86 and/or CD206. To calculate these values, the following formula was used:

(5) $${\%\;\mathrm{PFCE}-\mathrm{NE}}^{+/-}/{\mathrm{CDn}}^{+}\;\mathrm{cells}\;=\left(\frac{{\mathrm{number}\;\mathrm{of}\;\mathrm{PFCE}-\mathrm{NE}}^{+/-}{/\mathrm{CDn}}^{+}\mathrm{cells}\;}{\mathrm{total}\;\mathrm{number}\;\mathrm{of}\;\mathrm{cells}}\right)*\;100$$

where *n* stands for 86 or 206. The mean values of the three images were then calculated.

### 2.12. Statistical Analysis

All data were presented as mean values ± standard error of the mean. Statistical analysis was carried out using GraphPad Prism 5.03 (San Diego, CA, USA). Statistically significant differences among experimental conditions were identified by applying one-way ANOVA or two-way ANOVA test, and Bonferroni or Turkey post hoc tests. *p*-values < 0.05, 0.01 and 0.001 were marked as *, ** and ***, respectively.

## 3. Results

### 3.1. PFCE-NE and Liposome Characterization 

The PFCE-NE, prepared by sonication, consisted of particles with a mean hydrodynamic diameter of 135 ± 15 nm and a polydispersity index (PDI) of 0.12 ± 0.04. The ^19^F-NMR (Nuclear Magnetic Resonance) signal was characterized by a single peak located at −15.3 ppm with respect to TFA. Longitudinal (T_1_) and transverse (T_2_) relaxation times, determined at 25 °C and 7 T, were 800 ± 12 and 290 ± 8 ms, respectively. 

### 3.2. In Vitro Experiments 

**Figure 2:**
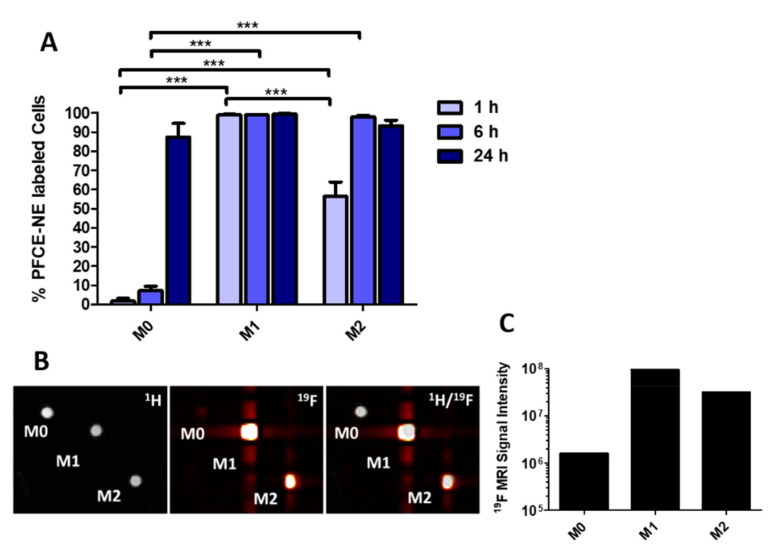
(**A**) Percentage of perfluoro-15-crown-5 ether (PFCE)-based nanoemulsion (PFCE-NE) positive cells detected by confocal microscopy in M0 non-polarized macrophages and in M1 and M2 polarized cells. Cells were incubated with PFCE-NE for 1, 6, and 24 h. Two-way ANOVA test, Bonferroni post-hoc test, *** *p* < 0.001. (**B**) ^1^H, ^19^F, and ^1^H/^19^F MRI of a cell phantom containing M0, M1, and M2 cells incubated with 2 mM of PFCE-NE for 1 h and (**C**) corresponding ^19^F signal intensity determined by ^19^F MRI.

In vitro experiments were performed to assess the PFCE-NE uptake in three different primary macrophage populations (namely M0, M1, and M2) and to evaluate whether the nanoparticles could induce macrophage polarization. Three different incubation time points (1 h, 6 h, and 24 h) have been evaluated. Each cell subset (M0, M1, and M2) displayed a different capability and kinetics to incorporate the nanoparticles (Figure 2A and Figure S1). M0 macrophages showed the slowest internalization rate since after a 6 h incubation with nanoparticles less than 10% (7.2 ± 2.2%) of cells were PFCE-NE-positive; at 24 h, the percentage significantly increased (87.3 ± 7.1%), but without reaching the labeling of the whole population. On the contrary, almost 100% of M1 macrophages were found PFCE-NE-positive already after 1 h incubation, with this percentage remaining stable for the whole observation period until 24 h. Finally, the uptake rate of M2 macrophages increased during the time, but more slowly as compared to M1: the half population was labeled after 1 h (56.3 ± 7.6%), whereas almost 100% of cells showed PFCE-NE-positivity only after 6 h of incubation; this level of internalization was approximately maintained until 24 h (93.3 ± 2.9%). Consequently, the highest uptake rate was observed in M1 cells, as the internalization measured after 1 h exposure was already statistically different as compared to that of the other groups (*p* < 0.001). ^19^F MRI of M0, M1, and M2 cells incubated with PFCE-NE for 1 h perfectly matched the results reported in Figure 2A, with the highest fluorine signal detectable in M1 cells and a clear spot visible in M2 cells. Instead, in M0 cells the signal was hardly detectable (Figure 2B,C). 

**Figure 3:**
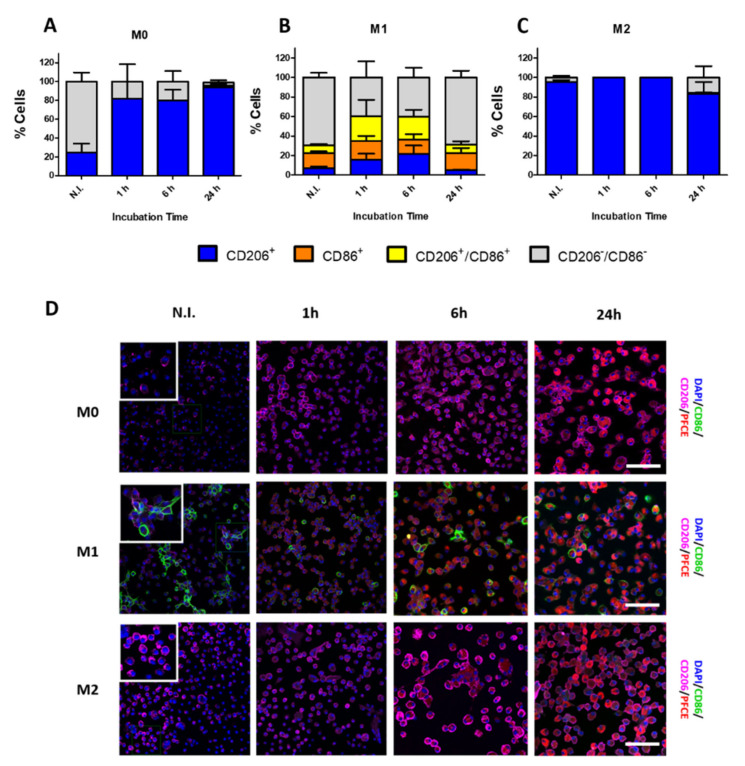
PFCE-NE impact on cell polarization displayed as the percentage of cells positive/negative to CD86 and/or CD206 in M0 (**A**), M1 (**B**), and M2 (**C**) cells. The percentage of cells was calculated on the total number of cells in N.I. condition or on PFCE-NE positive cells in incubated cells. Detailed statistical analysis can be found in Figure S3 (two-way ANOVA test, Bonferroni post hoc test). (**D**) Representative images of M0, M1, and M2 cells before (N.I.) and 1, 6, and 24 h after incubation with PFCE-NE. Cell nuclei are displayed in blue (DAPI), rhodamine of PFCE-NE in red, CD206 in magenta, and CD86 in green. Images were acquired with the confocal microscope. Scale bar = 100 μm.

Subsequently, the impact of PFCE-NE on cell polarization was investigated in the different cell populations. Both cell morphology and specific markers expression were examined before and at different times after incubation with PFCE-NE. According to the literature [27], M1 and M2 populations were respectively identified by immuno-recognition via anti-CD86 and anti-CD206 antibodies. Four different patterns were observed: CD206^+^ cells (M2 macrophages), CD86^+^ cells (M1 macrophages), CD86^+^/CD206^+^ cells (presumably M1/M2 mixed cell-phenotype) [28,29], and CD86^−^/CD206^−^ cells (presumably naïve M0 macrophages). Before the incubation, M0 macrophages were mostly CD86^−^/CD206^−^, with a certain fraction (about 20%) of CD206^+^ (Figure S2). In the M1 population, ≈15% of the cells were positive to CD86 only, whereas the majority appeared to be negative for both markers and a limited percentage (7.7 ± 1.5%) was positive for both of them. However, it is important to notice that this was the only population to show positivity for CD86^+^, similarly to what is reported in the literature [30]. Finally, almost the totality of M2 cells (95.6 ± 1.9%) appeared CD206^+^, while the remaining part was negative for both markers considered. After 1 h of incubation, around 80% of M0 macrophages showed a significantly higher positivity for CD206 compared to the non-incubated (N.I.) ones, while the remaining fraction was negative for both markers; similar data were calculated for 6 and 24 h post-incubation (≈80 and 94%, respectively) (Figure 3A and Figure S3A). Even if displaying the most heterogeneous cellular subset profile, M1 demonstrated the most balanced uptake rate through the various time points. Whereas the CD86^+^ fraction remained quite stable and comparable to N.I. condition (about 15–20%) at all the time-points, the percentage of CD206 marker expression (alone or in combination with CD86) strongly increased during 6 h of incubation. However, 24 h post-incubation the distribution of the four subsets matched again the subset profile of N.I. control cells (Figure 3B and Figure S3B). Finally, M2 macrophages, similarly to the trend seen in N.I. condition, at 1 h and 6 h post-incubation were all CD206^+^ (100.0%). However, at 24 h, some cells (7.5 ± 6.9%) were found positive to both CD206 and CD86, whereas the CD206^+^/CD86^−^ cell fraction significantly decreased to around 83% (Figure 3C and Figure S3C). Representative images of cell labeling with PFCE-NE and cell staining with anti-CD206 and anti-CD86 antibodies are displayed in Figure 3D. 

**Figure 4:**
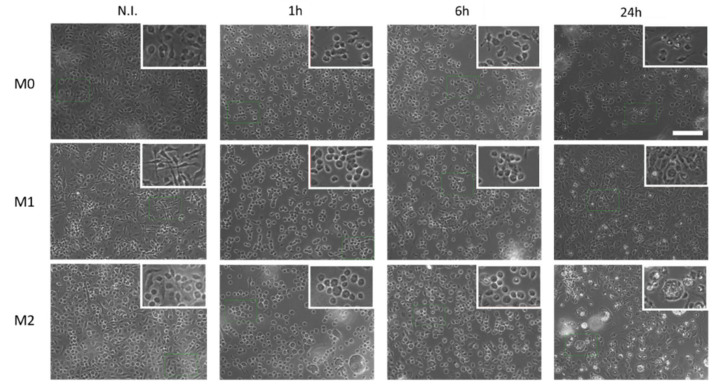
Phase-contrast images of M0, M1, and M2 macrophages before (N.I.) and 1, 6, or 24 h after PFCE-NE incubation. The insets show a zoom of the reported pictures (zoom factor 1.9), to better appreciate cell morphology. Scale bar = 100 µm.

Moreover, the effects of PFCE-NE on macrophage morphology after different times of incubation were also qualitatively assessed (Figure 4). In N.I. condition, M0 cells mostly showed a round and small morphology with few cells displaying amoeboid or elongated shape. M1 macrophages presented a peculiar morphology, with an elongated cell body due to characteristic cytoplasmic extension on the cellular surface. In the M2 population, cells displayed a heterogeneous morphology, including roundish or elongated cell bodies (“spindeloid” macrophages). Moreover, several multinucleated giant cells [31] were detected. After 1 h incubation with PFCE-NE, in all three macrophage populations, cells presented a roundish morphology. M1 macrophages lost the typical cytoplasmic extensions and, in M2 macrophages, the presence of multinucleated giant cells increased. At 6 h post-incubation, the predominantly observed morphology was still round, but cytoplasmic extensions started to appear on the M0 cell body, becoming even more diffuse at 24 h post-incubation. In M1 cells, at 24 h post-incubation, a mostly elongated shape prevailed, while in M2 cells the shape was still round, but multinucleated cells became more visible. In summary, these qualitative observations revealed that PFCE-NE affected the macrophage morphology, especially in the first hours of incubation.

### 3.3. Blood Half-Life Time Determination by ^19^F MRS

**Figure 5:**
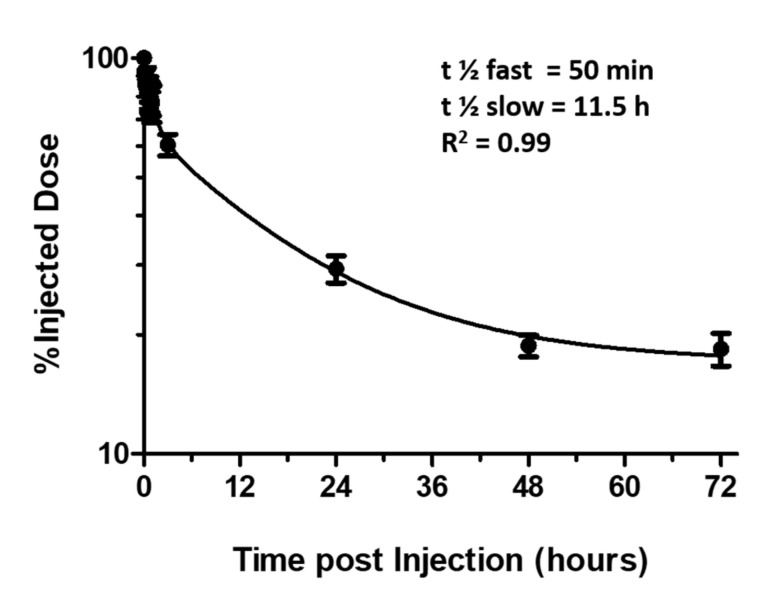
Blood half-life time calculation of PFCE-NE in healthy mice carried out by ^19^F MRS (*n* = 3). Data points fitted a bi-phasic exponential decay (R^2^ = 0.99).

The blood half-life time of various fluorinated systems has been already evaluated in the past [16,32,33,34]. However, invasive techniques consisting of blood collection followed by ex-vivo ^19^F MRI/MRS were applied. The low sensitivity of the method required the collection of large blood volumes, reducing the sampling and the time window in which the blood clearance can be studied. Since no extravasation of fluorinated particles has been reported in the brains of healthy mice, by placing in the volume coil only the head of the animal, all the signal detected by ^19^F MRI/MRS should merely refer to the particles circulating in blood vessels (either free or sequestered by immune system cells, considering negligible the contribution of other tissues of the head). In this way, one can acquire a large amount of data, performing a precise sampling. After anesthesia, three healthy mice were accurately placed into the MRI scanner and after receiving 1 mmol/kg of PFCE-NE i.v., ^19^F MR spectra were recorded starting from time 0 to 60 min and then at 3, 24, 48 and 72 h post-injection. The acquired spectra were integrated and the resulting values were expressed in terms of % injected dose and plotted against time (Figure 5) [34]. Data obtained were fitted with a bi-exponential fitting curve. Resulting blood half-life times were respectively t_1/2_ fast = 50 min and t_1/2_ slow = 11.5 h, R^2^ = 0.99.

### 3.4. ^19^F/^1^H MRI of Blood Samples

To prove monocyte/macrophage in vivo labeling, two healthy mice were i.v. administered with 1 mmol/kg PFCE-NE. Three hours post-injection, blood was collected from the vena cava inferior, heparinized, stratified via Ficoll–Histopaque method, and finally imaged by ^1^H/^19^F MRI. The fluorine signal localized mainly in the mononuclear cells ring already at three hours post-injection (Figure S4), thus indicating the occurrence of effective labeling in vivo.

### 3.5. In Vivo ^19^F MRI

Monocyte/macrophage infiltration at the lesion site was imaged by different ^1^H/^19^F MRI protocols. The advantage of using coupled ^1^H/^19^F MR imaging resides in an excellent degree of specificity due to the lack of any ^19^F background signal matched with the excellent spatial resolution provided by ^1^H acquisition, allowing to precisely locate the ^19^F signal. According to previously published protocols [35,36,37], ^19^F MRI was started 24 h post PFCE-NE administration, to allow the in vivo labeling of immune cells and subsequent recruitment of these cells at the injury site. In our study, various protocols already reported in the literature were slightly adapted and put into practice to compare the different information gained and select the best strategy to image this model. The amount of fluorine at the lesion site was quantified by ^19^F MRI after signal normalization using a 2 mM PFCE-NE suspension as reference. Since PFCE-NE were also expected to passively diffuse in inflamed areas via the endothelium because of their small size [37], the observed MRI signal was considered as a sum of the contribution of two phenomena, namely the free PFCE-NE diffusion and labeled macrophage recruitment. The MRI acquisition parameters used were always the same, with an imaging protocol lasting 1 h 20 min for MRI or 2 h if Point RESolved Spectroscopy (PRESS) was also carried out. Images acquired were mainly in the axial geometry, with a slice centered on the lesion (easily located by ^1^H MRI), one above, and one below it (3.0 mm thick per slice) (Figure S5). Images with sagittal geometry were occasionally acquired to highlight how the signal was mainly located at the lesion site; however, they were not routinely collected nor used for signal quantification as signal artifacts coming from the liver and blood vessels were often present (Figure S6). In images acquired with axial orientation, a strong signal coming from the liver could be noticed, as in liver sequestering of particles naturally occurs by RES system [38,39]. However, that signal was not quantified as it was beyond the scope of this work and required the acquisition of additional slices to include the whole organ volume.

### 3.6. Single Administration at 1 DPI, MRI at 2, 3, 5, 8, 11, and 14 DPI

**Figure 6:**
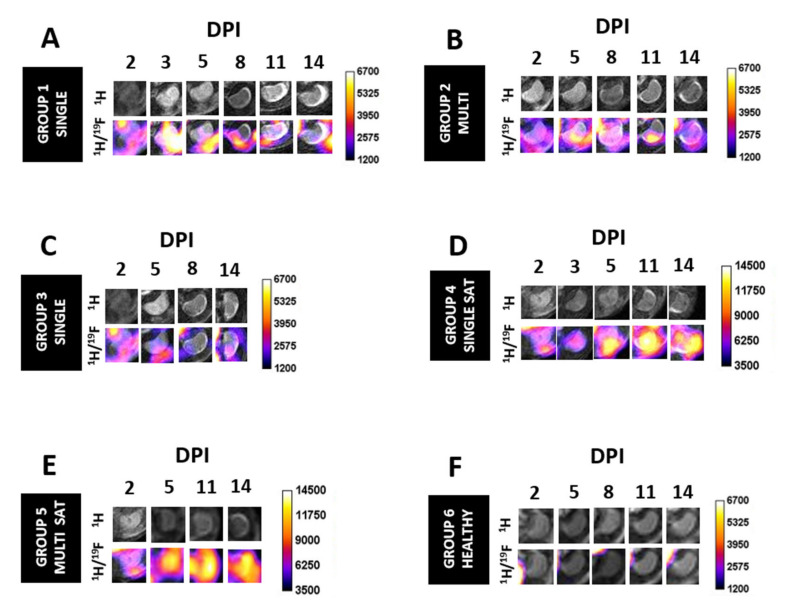
^1^H and ^1^H/^19^F MR images of the spinal cord at the lesion level following different protocols of PFCE-NE administration: (**A**) spinal cord injury *(SCI) mice, single administration of PFCE-NE at 1 days post-injury (DPI); (**B**) SCI mice, multiple administration of PFCE-NE at 1, 4, 7, 10, and 13 DPI; (**C**) SCI mice, single administration of PFCE-NE at 1, 4, 7, or 13 DPI; (**D**) SCI mice, single administration of PFCE-NE at 1 DPI with pre-saturation of Kupffer cells; (**E**) SCI mice, multiple administration of PFCE-NE at 1, 4, 7, 10, and 13 DPI with pre-saturation of Kupffer cells; (**F**) healthy mice, multiple administration of PFCE-NE at day 1, 4, 7, 10, and 13 post-enrollment.

**Figure 7:**
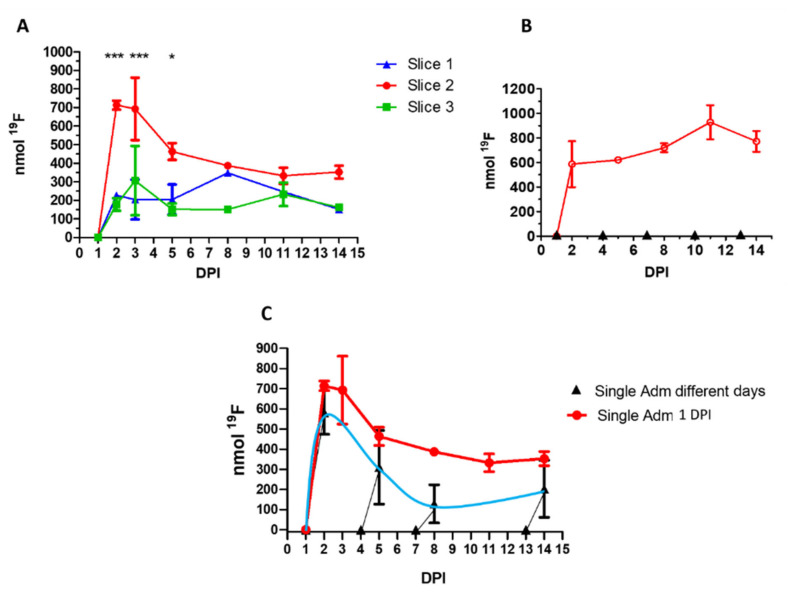
(**A**) Quantification of fluorine content (nmol ^19^F) by ^19^F MRI at the lesion site (Slice 2) or above (slice 1) and below (slice 3) following a single administration of PFCE-NE at 1 DPI. MRI was carried out at 2, 3, 5, 8, 11, and 14 DPI (*** *p* < 0.001, * *p* < 0.05 two-way ANOVA test, Bonferroni post-hoc test, *n* = 3). (**B**) Quantification of fluorine content by ^19^F MRI at the lesion site following multiple administrations of PFCE-NE (1, 4, 7, 10, 13 DPI, indicated by black triangles). MRI was carried out at 2, 5, 8, 11, and 14 DPI. No statistically significant differences are present among the signal quantified at different DPI (*n* = 3, one-way ANOVA Test, *p* > 0.05). (**C**) Comparison between the signal quantification obtained following one administration of PFCE-NE at 1 DPI, followed by MRI monitoring at 2, 3, 5, 8, 11, and 14 DPI (red circles) and the signal obtained after single administrations of PFCE-NE at 1, 4, 7 or 13 DPI (black triangles, blue light line) (*n* = 6, two-way ANOVA Test, *p* > 0.05).

First, we performed a single PFCE-NE administration 24 h post-injury and sequential ^1^H/^19^F MRI sessions on the same animals at 2, 3, 5, 8, 11, and 14 DPI (Figure 1A). This protocol has been already adopted to track macrophages in Inflammatory Bowel Disease (IBD) [18], tumor [35], arthritis [19], and myocardial infarction [16], and has the advantage of requiring only one administration of the suspension to track cell recruitment in inflamed regions also for a long period of time (3 days for tumor, 7 days for myocardial infarction, 30 days for IBD). However, as after three days PFCE-NE available in the blood is absent or limited [16], the MRI information gained from 5 DPI might be linked uniquely to already recruited macrophages. Here, a statistically significant accumulation of fluorine at the lesion site was observed (Slice 2) in comparison to regions above (Slice 1) or below (Slice 3) injury (Figure 6A and Figure 7A) at 2, 3, and 5 DPI, confirming that the recruitment is mainly restricted to the injured site. In the imaging volume associated with injury (slice 2), strong recruitment of PFCE-NE-labeled cells occurred 2 DPI and started to decrease only from 3 DPI, with a slight increase at 14 DPI.

### 3.7. Multiple Administrations at 1, 4, 7, 10, 13 DPI, MRI at 2, 5, 8, 11, and 14 DPI

Then PFCE-NE was administered multiple times starting from 1 DPI every three days to compensate for the clearance of PFCE-NE from the blood, and MRI was performed 24 h after each injection (Figure 1B, Figure 6B). As displayed in Figure 7A, the signal tends to decrease 2 days post-injection, without ever becoming null, so as that the contribution of different administrations in the signal at each time point resulted arduous to identify. In Figure 7B, fluorine quantification at the lesion site only is reported, showing higher values than in the case of a single administration, with maximal accumulation at 11 DPI (928.2 ± 128.2 nmol, fourth administration).

### 3.8. Single Administration at 1, 4, 7, or 13 DPI, MRI at 2, 5, 8, or 14 DPI

To correlate the acquired signal with variations of the inflammation states at different time points after tissue damage, a single administration of PFCE-NE was performed in various animals at different DPI, followed by MRI (24 h post-injection) and sacrifice for ex-vivo studies (Figure 1C). As shown in Figure 6C and Figure 7C, the signal intensity is highly variable over time, even if representative of a single PFCE dosage. The higher signal (corresponding to 581.6 ± 108.4 nmol ^19^F) was detected 2 DPI. The signal then progressively decreased at 5 and 8 DPI (≈300 and 130 nmol, respectively) to slightly increase again at 14 DPI (≈200 nmol). Surprisingly, this trend matched the one observed after a single administration at 1 DPI, followed by MRI acquisitions at different DPI (first protocol tested).

### 3.9. Single Administration at 1 DPI with Saturation of Kupffer Cells, MRI at 2, 3, 5, 11, and 14 DPI

**Figure 8:**
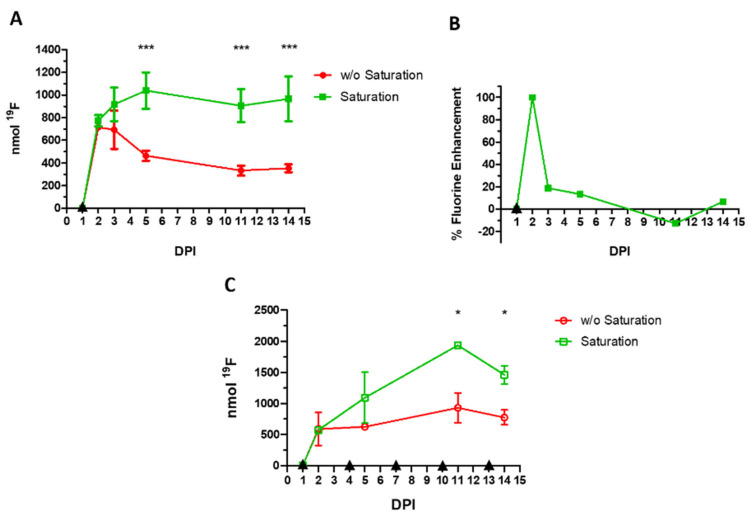
(**A**) Quantification of fluorine content by ^19^F magnetic resonance imaging (MRI) at the lesion site following a single administration of PFCE-NE at 1 DPI, preceded (green squares) or not (red circles) by liposome injection (volume = 200 µL) to saturate Kupffer cells. MRI was carried out at 2, 3, 5, 11, and 14 DPI. *** *p* < 0.001, two-way ANOVA Test, Bonferroni post-hoc Test, *n* = 6. (**B**) % Fluorine enhancement calculated over each previous MRI session in mice administered with both liposomes and PFCE-NE at 1 DPI. (**C**) Quantification of fluorine content by ^19^F MRI at the lesion site following multiple administrations of PFCE-NE (1, 4, 7, 10, 13 DPI) preceded (green open squares) or not (red open circles) by liposome injection to pre-saturate liver Kupffer Cells. MRI was carried out at 2, 5, 11, and 14 DPI. * *p* < 0.05, two-way ANOVA Test, Bonferroni post-hoc test, *n* = 6. Black triangles correspond to PFCE-NE with or *w/o* liposome injection.

To increase the PFCE-NE delivery at the target site, lipidic vesicles were administered to mice 10 min before the PFCE-NE injection to pre-saturate liver Kupffer cells, which, as part of the RES, are known to sequester a high amount of PFCE-NE [40,41]. MRI was then performed 24 h later and at 3, 5, 11, and 14 DPI (Figure 1D, Figure 6D). By pre-injecting liposomes in healthy mice, available PFCE-NE was significantly higher only in the first hours post-injection (82.3 vs. 60.4% of the injected dose, 3 h post PFCE-NE injection, *p* < 0.05, Figure S7), but not in the following hours and days. No differences were found in blood half-life times. In injured mice (Figure 8A), pre-administration of lipidic vesicles effectively resulted in a dramatically stronger signal in the lesion site, resulting in more than two-fold higher than that measured after single administration without pre-saturation, with a peak at 5 DPI. (1038.4 ± 160.6 nmol with saturation, 463.3 ± 45.5 nmol *w/o* saturation, *p* < 0.05). The signal then slowly decreased at 11 DPI (904.8 ± 145.8 nmol with saturation, 332.5 ± 44.1 nmol *w/o* saturation, *p* < 0.05), to increase again at 4 DPI (965.5 ± 198.5 nmol with saturation, 352.5 ± 34.9 nmol *w/o* saturation, *p* < 0.05). The peak of signal intensity was reached at 5 DPI, whereas in previous experiments it occurred at 2 DPI. Hypothesizing that through pre-saturation a higher amount of PFCE-NE is available for monocyte/macrophage labeling, the fluorine signal enhancement in the lesion site, calculated as a percentage over the previous MRI acquisition at each day, resulted in a signal trend that matched that one reported for protocol 1 and 3 (Figure 8B). 

### 3.10. Multiple Administrations at 1, 4, 7, 10, 13 DPI, with Saturation of Kupffer Cells, MRI at 2, 5, 11, and 14 DPI

Multiple administrations of PFCE-NE (1, 4, 7, 10, and 13 DPI), always preceded by lipidic vesicle injection, and followed by MRI (at 2, 8, 11, and 14 DPI) were performed (Figure 1E, Figure 6E). Results obtained displayed an extraordinarily high accumulation of fluorine in the injured site at 11 DPI (1934.0 ± 8.3 nmol with pre-saturation, 928.2 ± 139.2 nmol *w/o* pre-saturation, *p* < 0.05), being around 170% higher than the maximum accumulation obtained with a single administration without saturation (Figure 8C). 

### 3.11. Healthy Mice

To exclude the extravasation of PFCE-NE in absence of injury, the nanoemulsion was also administered to healthy mice (Figure 1F): no signal was detected in neither the spinal cord nor the brain (data confirmed also by ex-vivo histological studies, Figure S8). No extravasation of neutral nanosystems with a size of 100-200 nm, in fact, is expected in the CNS in healthy subjects [42,43]. However, a strong signal was visible in the liver due to RES uptake. A comparison between the MRI signal in the liver and spinal cord of s SCI and a healthy mouse is displayed in Figure S9.

### 3.12. PRESS

**Figure 9:**
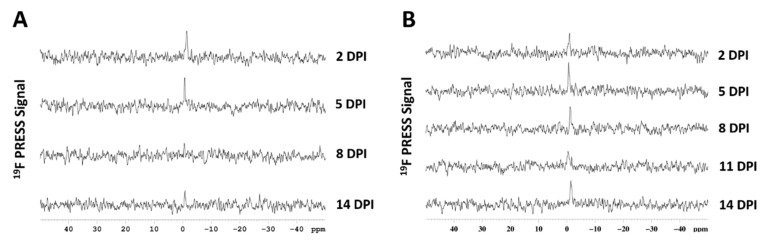
^19^F Point Resolved Spectroscopy (PRESS) of SCI acquired at different days post-injury following (**A**) single administrations of PFCE-NE 24 h before MRI (Figure 1C) or (**B**) multiple administrations of PFCE-NE every three days (Figure 1B).

As an alternative to ^19^F MRI, the possibility of performing ^19^F-MRS at the site of injury using PRESS sequence was also investigated. Localized ^1^H shim was carried out in a 3.5 × 3.5 × 3.5 mm^3^ voxel located exactly in the site of injury, then ^19^F-PRESS was performed. In Figure 9, spectra obtained at different DPI following single PFCE-NE administrations at different time points (Figure 1C, Figure 9A) or multiple administrations of the fluorinated contrast agent every three days (Figure 1B, Figure 9B) are displayed. In principle, this technique can be used as an alternative to the imaging ^19^F MR setup, being faster (around 20 min vs. around 1 h), even if for an accurate signal quantification the acquisition of another spectrum (reference solution) is required. Furthermore, the information about possible infiltration of macrophages in regions adjacent to the inflamed one cannot be gained. 

### 3.13. Ex-Vivo Immunofluorescence Analyses

**Figure 10:**
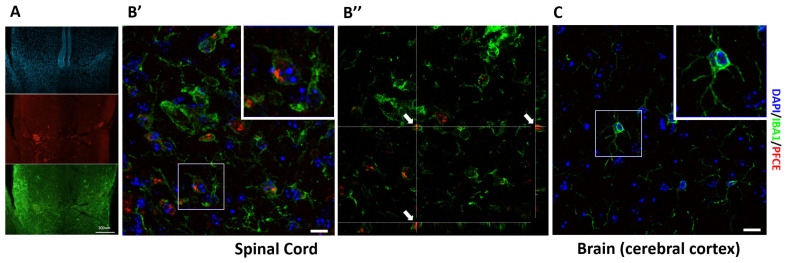
(**A**) Fluorescence microscopy images of the injured spinal cord at the lesion level at 14 DPI following a single administration of PFCE-NE. (**B’**) Confocal fluorescence microscopy at the lesion level of the spinal cord and (**B”**) rotations along the *x*- and *y*-axes showing the internalization of PFCE-NE (red) into the IBA1-positive macrophage (green) on the *z*-axis (arrows) (same field of acquisition of B’). (**C**) Confocal fluorescence microscopy at the brain level (cerebral cortex), 14 DPI following a single administration of PFCE-NE. Nuclei are displayed in blue. Scale bar = 200 µm in A, 10 µm in B’, 20 µm in C.

To corroborate the in vivo data, the presence of rhodamine PFCE-NE in dissected injured spinal cords was verified by histological analyses. As displayed in Figure 10, the PFCE-NE was clearly visible in the spinal cord: a remarkable number of particles was detectable at the lesion site, phagocytized by IBA1-positive microglia and macrophages, whereas PFCE-NE-positive cells were only sporadically observed at the rostral and caudal level or in the brain cortex. 

**Figure 11:**
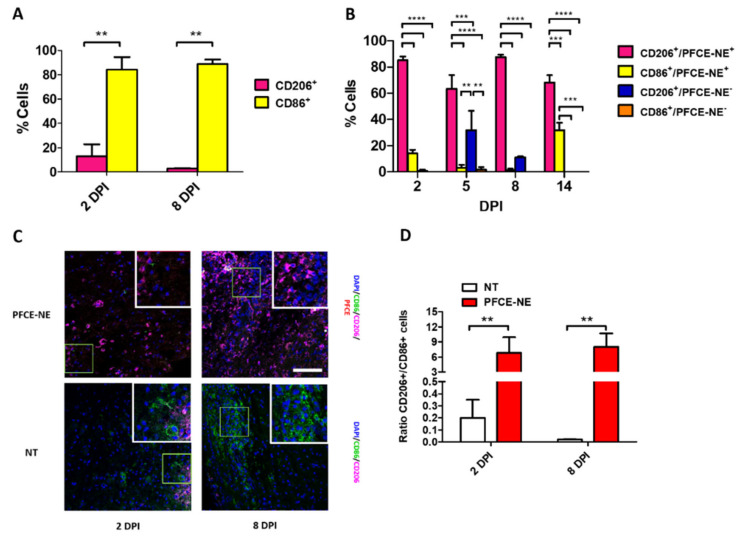
(**A**) Percentage of M1 (CD86^+^) and M2 (CD206^+^) cell populations at 2 or 8 DPI calculated at the lesion site in mice not treated (NT) with PFCE-NE. Two-way ANOVA test, Tukey post-hoc test ** *p* < 0.01. (**B**) Percentage of M1 (CD86^+^) and M2 (CD206^+^) cell populations, either positive or negative for PFCE-NE, calculated at the lesion site. Percentages were calculated on the total number of cells. Two-way ANOVA test, Tukey post-hoc test ** *p* < 0.01, *** *p* < 0.001, **** *p* < 0.0001. Statistical analysis between different days post-injection can be found in SI (Figure S10). (**C**) Representative images acquired at 2 or 8 DPI by confocal microscopy in SCI injured mice treated or not with PFCE-NE. Scale bar 100 µm. (**D**) Ratio between CD206+ and CD86+ cells calculated in SCI at 2 and 8 DPI in NT and PFCE-NE treated mice. Two-way ANOVA test, ** *p* < 0.01.

To verify whether PFCE-NE could affect macrophage polarization also in vivo, the positivity of immune cells to CD86 and CD206 was assessed to discriminate M1 and M2 phenotypes, respectively. Then, results from confocal microscopy analysis were correlated to ^19^F MRI data. Even if M1/M2 polarization of macrophages in untreated SCI mice has been already largely investigated and reported in the literature [30], the positivity of immune cells to CD86 and CD206 in our model at 2 and 8 DPI was checked. As expected, most of the phagocytic cells were predominantly CD86+ (M1 phenotype) at both time points, with less than 13% and 3% of CD206+ cells at 2 and 8 DPI, respectively (Figure 11A,C). In animals receiving a single PFCE-NE administration 24 h before MRI and sacrifice (group 3, Figure 1C), instead, the majority of the macrophages (both M1 and M2) were PFCE-NE-positive, in a range varying between 65% and 100% (respectively, at 5 and 14 DPI) (Figure 11B,C). These data confirmed not only that in vivo the macrophages largely internalized the PFCE-NE, but also that the signal visualized in ^19^F MRI actually corresponded to the macrophage population in the lesion site. Notably, among the PFCE-NE-positive cells, the majority was CD206^+^. In particular, the number of CD206^+^/PFCE-NE^+^ M2 cells was very high at all the time points (ranging from 63.33% ± 14.90 at 5 DPI to 87.53% ± 2.75 at 8 DPI). Instead, the percentage of PFCE-NE-positive M1 macrophages (CD86^+^/PFCE-NE^+^) reached the highest number at 14 DPI (31.91% ± 7.91), whereas at the other time-points it did not exceed 15% (Figure 11B and Figure S10). The ratio between CD206+ and CD86 + cells at 2 and 8 DPI in untreated and PFCE-NE treated mice is displayed in Figure 11D. 

Next, the reliability of the signal observed in ^19^F MRI was further verified on spinal cord sections of injured mice. The average number of M1 and M2 PFCE-NE positive cells per mice, at different DPI, was calculated (Figure S11). Despite the variability and the limited number of animals at some time-points, the counts highlighted a trend similar to those observed by MRI (Figure 7C), with a peak of cells in the first days (in particular at 5 DPI), followed by a reduction in the macrophage number, and then a new increase at 14 DPI (one-way ANOVA test, Tukey post-hoc test, not significant results).

**Figure 12:**
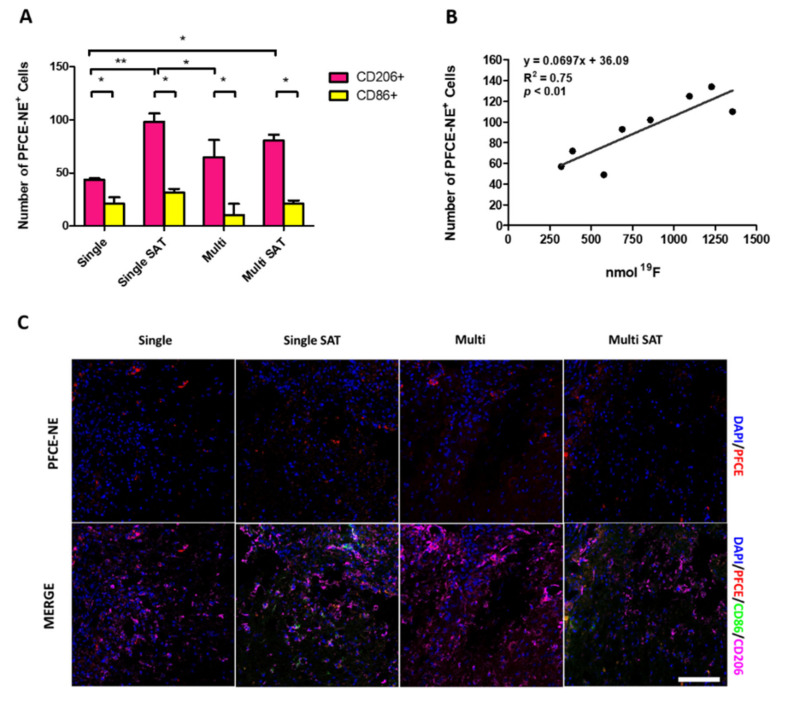
(**A**) Counts summarizing the average number of PFCE-NE-positive cells (both M1 and M2) at 14 DPI in case of multiple (Multi) or single PFCE-NE administration with or without saturation (SAT). Two-way ANOVA Test, * *p* < 0.05, ** *p* < 0.01, *n* = 8. (**B**) Correlation between the mean number of PFCE-NE^+^ M1/M2 cells found near the lesion site and the number of ^19^F nmol measured by MRI in the same region. Linear regression, R^2^ = 0.75, ** *p* < 0.01, *n* = 8. (**C**) Immunofluorescence reactions to evaluate CD86 and CD206 labeling in longitudinal sections of the spinal cord at 14 DPI. Images were acquired near the lesion site and different patterns can be observed in the macrophages’ subset. Scale bar = 100 μm.

Finally, a comparison among the mean number of CD206^+^/or CD86^+^/PFCE-NE^+^ cells found in ex-vivo slices at 14 DPI according to the protocol adopted (single or multi-administration, with or without saturation) was carried out. Moreover, the mean number of PFCE-NE^+^ cells in the injured site and the amount of fluorine-related signal measured at the lesion site were correlated for each analyzed mouse. As displayed in Figure 12A, in all protocols tested, at 14 DPI the majority of PFCE-NE^+^ cells is CD206^+^ suggesting the prevalence of an M2 phenotype. The correlation displayed in Figure 12B between the mean number of all PFCE-NE^+^ cells found in examined ex-vivo slices and the nmol of ^19^F measured by MRI in the injured site proved to be statistically significant (*p* = 0.0053), with an R^2^ value of 0.75, even if the number of samples is limited. In Figure 12C, images of ex-vivo immunofluorescence carried out on samples at 14 DPI are reported, while in Figure S12 the mean number of cells and corresponding mean nanomoles of ^19^F are displayed. These results proved for the first time the extremely high reliability of ^19^F MRI signal to track macrophage recruitment at the lesion site.

## 4. Discussion

PFC-based nanosystems have been widely used to track selected populations of cells, due to their unambiguous signal in MRI/MRS. Main applications include labeling of T cells [44], dendritic cells [45], and stem cells [46,47]. Extensive studies demonstrate their low toxicity profile, even at very high doses, with a median lethal dose (LD50) ranging from 30–41 g PFC/kg body weight [48]. Moreover, the lack of effects over the cell phenotype and function after intracellular labeling has been reported in dendritic, T, and B cells [24]. However, the effects over macrophage phenotype have never been described, even though such aspect can be of utmost relevance given that these systems are often used to in vivo label monocytes/macrophage and track/monitor inflammation [21,37]. Being carried in the inflamed sites by immune cells, they might exert impactful effects on the inflammation theater. In the present work, a PFCE-based NE has been prepared and tested both in vitro and in vivo. The mean size of particles (≈135 nm) was below the threshold size indicated as possibly affecting the cell immunological status (500 nm) [26]. The incubation of M0, M1, and M2 cells with PFCE-NE displayed higher and faster uptake by M1 cells, as reported in the literature [49,50] with almost 100% cells loaded with PFCE-NE already after 1 h of incubation. M2 cells, instead, reached 100% of PFCE-NE uptake after 6 h of incubation, while M0 cells showed the lowest uptake rate with around 90% cells loaded with PFCE-NE only after 24 h of incubation. However, as expected, all the populations displayed a strong avidity towards the fluorinated system. Even if the effective cell internalization of PFCE-NE was not proved by electron microscopy in our experiments, previous studies demonstrated the localization of this nanosystem in the cell cytoplasm of dendritic cells, visible as smooth spheroids, after 24 h of incubation [26]. Moreover, the high lipophobic and hydrophobic nature of this compound prevents its incorporation into cell membranes, favoring a phagocytic/endocytic pathway [51,52,53]. Our confocal acquisitions confirm the PFCE-NE internalization in the macrophages too (Figure 3 and Figure 10). While fluorine-labeled DCs and T lymphocytes were reported to remain unaltered in their profile [24], in our study, upon PFCE-NE internalization bone marrow-derived macrophages revealed significant changes in the marker expression especially at early time points (1 and 6 h). PFCE-NE-internalizing M0 were for the most part positive for CD206 (CD206^+^/PFCE-NE^+^) whereas only a small fraction of the population remained negative for both CD206 and CD86 (Figure 3 and Figure S3). Moreover, in M1 cells a slight, but significant, increase in CD206 expression was observed, alone or in combination with CD86. CD206^+^/CD86^+^ cells should represent a mixed M1/M2 cell population that is switching from M1 phenotype (pro-inflammatory) to M2 phenotype (anti-inflammatory) [51,54,55]. In M2 cells the expression of CD206 was unaffected (Figure 3 and Figure S3), only showing a significant decrease at 24 h post-incubation, but remaining included in a range of high expression values (from 100% to 83%). Cell morphology changed in all three populations in response to PFCE-NE incubation: in particular, M1 cells switched from an elongated to a roundish shape, and in M2 cells the number of multinucleated giant cells definitely increased. Overall, the in vitro experiments demonstrated that PFCE-NE is easily internalized by macrophages and can direct their cell polarity towards an anti-inflammatory (M2) profile, with the strongest effects observed in M0 and M1 populations at the earliest time-points (1h and 6h).

Based on these results, we wondered whether PFCE-NE was also able to modulate the macrophage phenotype in vivo, in addition to allow inflammation monitoring by ^19^F MRI, thus potentially exerting a double function, known as theranostic [56]. Mice were sacrificed at different time points (Figure 1C) and the spinal cords were dissected to perform ex-vivo immunofluorescence. First, the minimum measured PFCE-NE uptake in macrophages was 65% at all the time-points, confirming the capability of macrophages to internalize fluorinated nanoparticles in vivo (Figure 11B). Evidence in the literature already showed that an M1-to-M2 transition can occur in vivo in SCI murine models in response to modulation of the environmental stimuli affecting macrophages (for example, by modifying the effect of microglia with neurotrophic factors or by modulating cytokines and chemokines production at the lesion site) [30,57]. Nevertheless, thus far, the effects of PFCE-NE on macrophage polarization in vivo have never been extensively studied. According to the literature, at 2 and 8 DPI, we observed a high number of CD86-positive M1 cells (Figure 11A). Interestingly, some studies suggest an extensive M1-to-M2 switch after the injection of poly(lactide-co-glycolide) (PLGA) nanoparticles [30]. Accordingly, in our experiments most of PFCE-NE^+^ cells appeared as positive for CD206, suggesting that indeed PFCE-NE can influence macrophage polarization towards an anti-inflammatory phenotype also in vivo. We then specifically evaluated ex vivo markers expression at 14 DPI to assess whether the different experimental strategies used brought to a different outcome in macrophage polarization, but we did not observe significant differences (Figure S13). 

Another relevant novelty reported in this paper is the estimation of blood clearance time of PFCE-NE. Half-life times of fluorinated nanosystems have been widely investigated in organs [34,38], but only data concerning fluorinated liposomes are reported as referred to the hematic residence time [32,33]. This lack can be justified by the high invasiveness of the measurement techniques, which requires massive blood collection. Nevertheless, in view of in vivo labeling procedures, it would be of utmost importance to know how long NPs are available in the blood for monocytes/macrophage labeling. Flögel et al. first displayed that PFCE-NE can be detected in blood in mononuclear cells for up to two days [16]. In our work, we investigated blood half-life time by in vivo MRS in healthy mice, acquiring sequential spectra of mouse brain from time 0 up to 72 h post PFCE-NE injection, assuming the absence of PFCE-NE extravasation in the central nervous system [58]. This technique has the advantage of allowing an assiduous sampling, for long time windows and without blood collection. However, it can be applied only to healthy mice where no extravasation of this kind of NPs is reported. The blood half-life time obtained for PFCE-NE displayed a bi-exponential decay with a fast t ½ of 50 min, followed by a slower decay with t ½ of 11.5 h, comparable to that reported for fluorinated liposomes (8.6–12.8 h) [58].

^19^F MRI was then employed to track immune cell recruitment in a model of SCI. Fluorinated compounds have been used several times to track inflammatory conditions, exploiting both ex-vivo or in vivo labeling [59]. However, especially for direct in vivo labeling, the protocols adopted in terms of the number of NPs administrations and days of follow-up are often discordant. In this work, we compared the information gained by applying the different administration/imaging protocols to identify the most suited procedure to study this kind of injury. In particular, we varied the number of PFCE-NE injections and the time between the injection and the MRI scan. Besides, the possibility of transiently and partially saturate Kupffer cells before PFCE-NE injection was investigated. The first protocol tested, the most used one, consisted of a single administration of PFCE-NE followed by MRI acquisitions carried out at different time points (Figure 1A). Results obtained displayed a steep increase in fluorine signal in the first days post-injury (Figure 7A), followed by a decrease in the following 10 days and a slight increase detected again at around 14 DPI (Figure 7A). Interestingly, the same trend was observed when single injections of PFCE-NE were carried out on different days (2, 5, 8, or 14 DPI, Figure 1C, Figure 7C). This observation suggests that data derived from both protocols reflect the occurrence of the two inflammatory waves reported in the literature in SCI [11,60]: the former at 2–3 dpi, when inflammation is proved to be stronger, the latter about 2 weeks or more after injury. A similar trend has been also observed ex vivo, by counting the macrophages at the lesion site (Figure S11). Such a trend presumably indicates that the recruitment of immune cells (in particular monocytes/macrophages) is not constant throughout the inflammatory response to SCI. Moreover, these data confirm that a therapeutic window between the acute phase and the chronic phase to treat SCI patients could be selected by ^19^F MRI [11]. A temporary decrease in the immune response at the lesion site might correspond to the optimal time to administer a treatment (drugs or stem cells) [61], promoting axonal regeneration [62] before the sub-acute/intermediate phase starts and the glial scar is formed [63].

The alternative protocol tested was already presented by Flögel et al. and Balducci et al. to monitor cerebral ischemia for 19 days [16] and collagen-induced arthritis for 29 days [20], respectively. It consisted of multiple administrations of PFCE-NE to the same animal, carried out every three days to compensate for the clearance of the particles from the bloodstream (Figure 1B). This protocol has the definite advantage of delivering a higher amount of contrast agent to the lesion site, a relevant aspect in case of co-administration of a drug or theranostic activity of the injected system. However, the multiple administrations complicate the quantification of the infiltrating monocytes/macrophages at different times post-injury. In fact, the decrease of the signal observed after each injection, which is necessary to calculate the signal enhancement, can be difficult to predict. In summary, this protocol might be helpful to follow qualitatively the process of infiltration of immune cells into the inflamed tissue and to deliver a high amount of contrast agent but displays limited efficacy in providing more quantitative data describing inflammation.

Another aspect investigated in this work was the possibility of delivering a higher amount of contrast agent at the lesion site by transiently and partially saturating Kupffer cells. This strategy has been previously reported by Liu et al. [7], to boost the efficiency of paclitaxel-loaded nanoparticles. However, while Liu et al. waited 90 min between the administration of liposomes and the theranostic agent, in this work just 10 min elapsed between the two administrations (Figure 1D). This time window was optimized according to pilot experiments performed in our lab (data not shown). As displayed in Figure 8, liposome pre-administration resulted in a significantly higher accumulation of fluorine in SCI (from +50 to +170%), with the saturation effect becoming evident starting from 48 h post PFCE-NE injection (3 DPI) [7], even if the effects over blood clearance time were maximal in the first three hours post-injection (Figure S7), with around 80% of the injected dose still circulating (in comparison to 60% in absence of saturation). However, albeit advantageous when delivering a drug or a theranostic agent, the saturation strategy might render the signal quantification and the selection of the optimal therapeutic window challenging. In the case of a single administration, the signal enhancement over the previous time point can be calculated at each day, reflecting the continuous recruitment of monocytes/macrophages over time. Two peaks were observed, at 2 and 14 DPI (Figure 8B), with a signal decrease occurring at around 8 DPI. This finding could make this method (namely, a single PFCE-NE administration with Kupffer cells pre-saturation) the optimal technique to monitor SCI, combining the possibilities to quantify the inflammation and deliver a high amount of contrast agent in one single approach. 

The MRI protocol adopted in this work required 1 h for the ^19^F MRI scan, in addition to 10–20 min for adjustments and ^1^H MRI. Even if such time is appropriate for routine clinical acquisition, even shorter acquisition times can be achieved by applying the latest generation MR sequences and data reconstruction methods [64]. An alternative approach could be the use of point resolved spectroscopy (localized in the injured site). Data acquired in the present work demonstrated the feasibility of using this method, lasting only 20 min; however, we encountered several issues in the quantification of the signal, probably due to the forced cubic shape of the acquisition voxel, which does not fit perfectly with mouse spinal cord anatomy. In our opinion, this method represents a valid and faster alternative to ^19^F MRI but in selected organs, such as the brain, where localized shim can be carried out easily and voxel size/shape can perfectly match the region of interest. However, it has to be taken into account that in PRESS, in contrast to MRI, the information about regions adjacent to the inflamed one cannot be acquired simultaneously. 

Finally, to verify that the signal measured in the SCI was effectively associated with infiltrating macrophages, the mean number of macrophages PFCE-NE^+^ observed in the lesion site by immunofluorescence was correlated with the nmol of ^19^F estimated at 14 DPI (Figure 12B). Interestingly, even if the count of macrophages only referred to a limited number of tissue sections, and the different nature of the detected signal (fluorescence vs. ^19^F-MRI) [65], a strong correlation (R^2^ = 0.75) between the MRI signal and ex-vivo results was discovered, confirming the specificity and reliability of the ^19^F MRI technique.

## 5. Conclusions

In conclusion, this work demonstrates that PFCE-NE can be used as contrast agents to non-invasively track the monocytes/macrophages infiltration in a SCI model by ^19^F-MRI and identify the most appropriate therapeutic time window. Moreover, the effects exerted by PFCE-NE on macrophage phenotype, with a prevailing switching towards the M2 (or alternative) profile, suggests a possible theranostic function of these nanoparticles, with tremendous potential future applications. Encouraging data in these regards were observed on treated mice. Nevertheless, dedicated and extensive behavioral tests are still required and will be performed in the future. Our comparative work enables the reader to select the most appropriate protocol according to the research objectives (quantitative data acquisition, visual monitoring of macrophage recruitment, theranostic purpose, rapid MRI acquisition, etc.). In the future, the possibility of predicting the outcome of possible therapeutic strategies by ^19^F MRI will be investigated. It has, however, to be considered that, through this technique, a huge amount of PFCE-NE was delivered to the liver (the organ signal was not quantified due to signal saturation with the sequence employed). Toxicity was not observed in our experiments; however, since a long in situ permanence time is expected [38], this aspect has to be carefully examined.

## Supplementary Materials

The following are available online at https://www.mdpi.com/article/10.3390/biomedicines9040379/s1.

## Abbreviations

AUC	Area under the Curve
b.w.	Body Weight
CNS	Central Nervous System
D1	Relaxation Delay
DC	Dendritic Cells
DLS	Dynamic Light Scattering
DMEM/F-12	Dulbecco’s Modified Eagle Medium/Nutrient Mixture F-12
DPI	Days Post Injury
DPPC	1.2-Dipalmitoyl-Sn-Glycero-3-Phosphocoline
DSPE-PEG2000	1.2 Distearoyl-Sn-Glycero-3-Phosphoethanolamine-N-(Methoxy (Polyethylene glycol)-2000) Ammonium Salt
EAE	Experimental allergic Encephalomyelitis
FBS	Fetal Bovine Serum
FOV	Field of View
i.v.	Intravenous
IBD	Inflammatory Bowel Disease
IFNγ	Interferon Gamma
IL-10	Interleukin 10
IL-4	Interleukin 4
LPS	Lipopolysaccharide
MRI	Magnetic Resonance Imaging
MRS	Magnetic Resonance Spectroscopy
N.I.	Non Incubated
NAV	Number of Averages
NDS	Normal Donkey Serum
NMR	Nuclear Magnetic Resonance
NS	Number of Scans
OCT	Optimal Cutting Temperature Compound
p.i.	Post Injury
P1	Pulse
PB	Phosphate Buffer
PBS	Phosphate Buffered Saline
PDI	Polydispersity Index
PFA	Paraformaldehyde
PFC	Perfluorocarbon
PFCE	Perfluoro-15-Crown-5 Ether
PFCE-NE	Perfluoro-15-Crown-5 Ether Based Nanoemulsion
PL1	Power Level
PRESS	Point Resolved Spectroscopy
RES	Reticuloendothelial System
RF	Rare Factor
Rhodamine-DOPE	1,2-Dioleoyl-Sn-Glycero-3-Phosphoethanolamine-N-(Lissamine Rhodamine B Sulfonyl) (Ammonium Salt)
ROI	Region Of Interest
RT	Room Temperature
SC	Spinal Cord
SI	Signal Intensity
SCI	Spinal Cord Injury
SW	Sweep Width
T_1_	Longitudinal Relaxation Time
T_2_	Transversal Relaxation Time
T_2w_	T_2_ Weighted
TE	Echo Time
TFA	Trifluoroacetic Acid
TR	Repetition Time
