# Supplementary material for: Imaging of Inflammation in Spinal Cord Injury: Novel Insights on the Usage of PFC-Based Contrast Agents
Source: Biomedicines. 2021 Apr 3;9(4):379. doi: 10.3390/biomedicines9040379 (PMC8065995; doi:10.3390/biomedicines9040379)
Supplement: Supplementary file 1 [file biomedicines-09-00379-s001.pdf]

Supplementary Information for

Imaging of Inflammation in Spinal Cord Injury: Novel Insights on the Usage of PFC-Based Contrast Agents

Francesca Garello [1,†] Marina Boido [2,3,4,*,†] Martina Miglietti [3] Valeria Bitonto [1] Marco Zenzola [1] Miriam Filippi [1] Francesca Arena [1,5] Lorena Consolino [1] Matilde Ghibaudi [2,3] Enzo Terreno [1,*]

1 Molecular and Preclinical Imaging Centers, Department of Molecular Biotechnology and Health Sciences, University of Torino, 10126 Torino, Italy; francesca.garello@unito.it (F.G.); valeria.bitonto@unito.it (V.B.); marcozenzola@gmail.com (M.Z.); filippimiriam@hotmail.com (M.F.); Francesca.Arena@bracco.com (F.A.); lconsolino@ukaachen.de (L.C.)
2 Department of Neuroscience "Rita Levi Montalcini", University of Turin, 10126 Turin, Italy; matilde.ghibaudi@unito.it
3 Neuroscience Institute Cavalieri Ottolenghi, University of Turin, 10043 Orbassano, Italy; martina.miglietti@edu.unito.it
4 National Institute of Neuroscience (INN), 10126 Turin, Italy
5 Institute of Biostructures and Bioimaging (IBB), Italian National Research Council (CNR), 10126 Torino, Italy
* Correspondence: marina.boido@unito.it (M.B.); enzo.terreno@unito.it (E.T.); Tel.: +39-(011)-6706613 (M.B.); +39-(011)-6706452 (E.T.)
† These authors contributed equally to this work.

This file includes: Figures S1 to S13

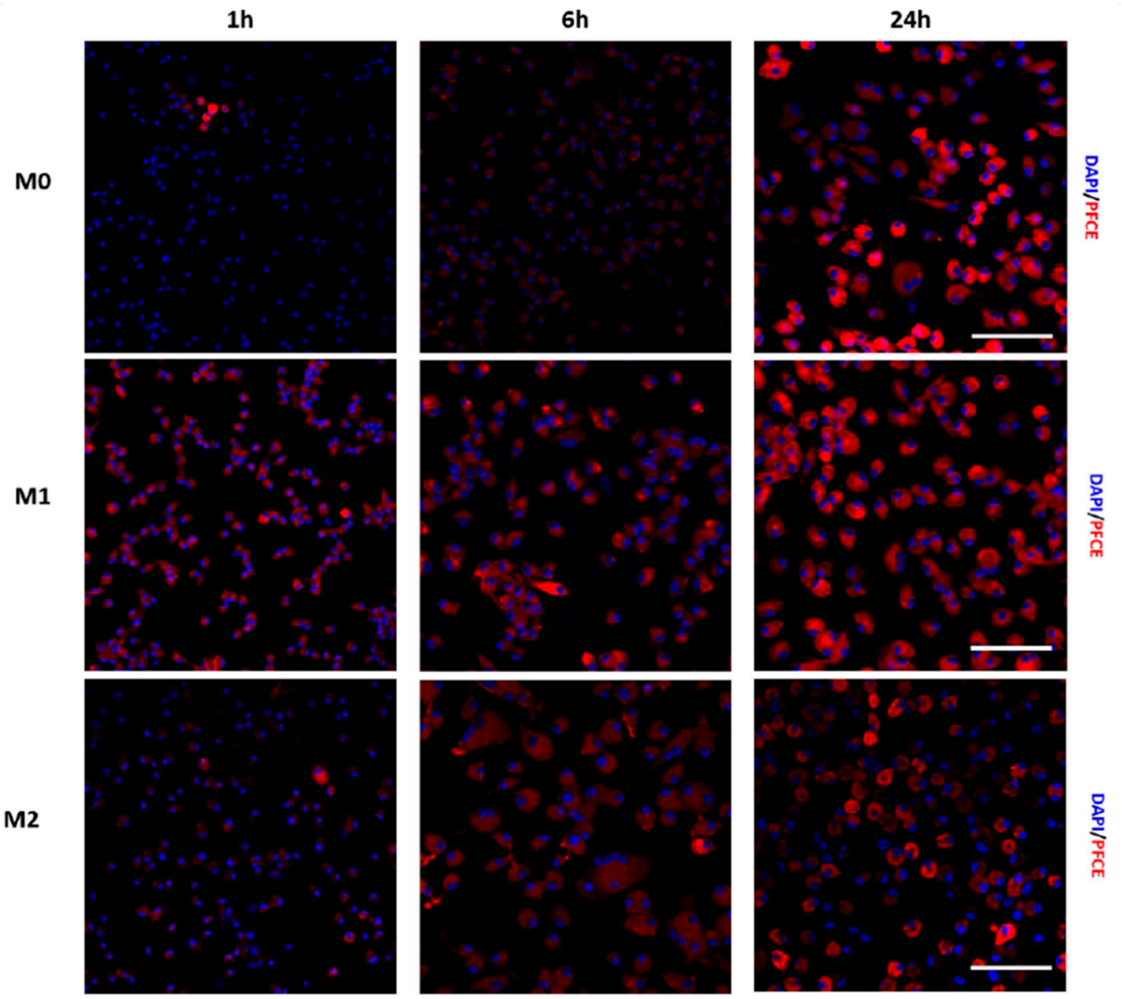

Figure S1. Representative images acquired with the confocal microscope of M0, M1 and M2 macrophages incubated with PFCE-NE for 1, 6 and 24 h. Scale bar = 100 μm. Cell nuclei are displayed in blue (DAPI), rhodamine of PFCE-NE is displayed in red.

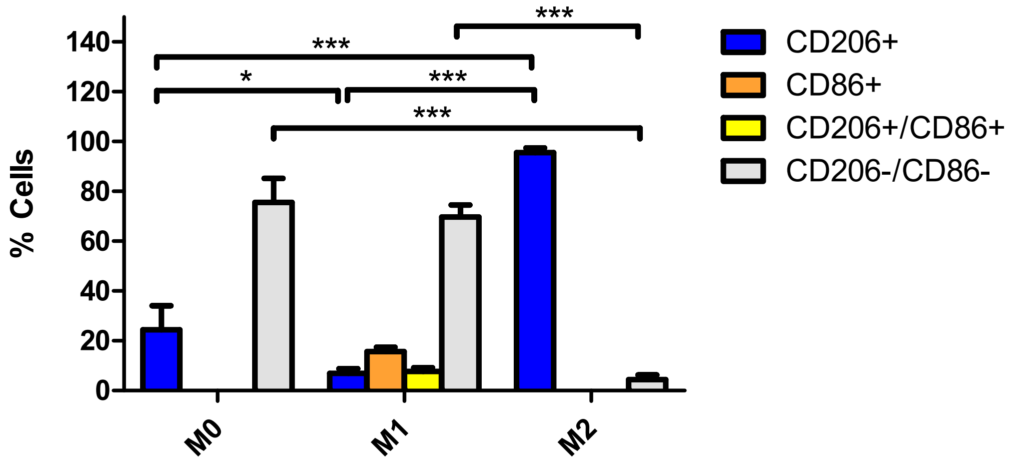

Figure S2. Percentage of cells CD206^{+}, CD86^{+}, CD206^{+}/CD86^{+} and CD206^{-}/CD86^{-} calculated over the total number of cells after cell polarization with LPS and IFN-γ (M1) or IL-4 and IL-10 (M2). Two-Way ANOVA test, Bonferroni post-hoc test (* $p < 0.05$, *** $p < 0.001$)

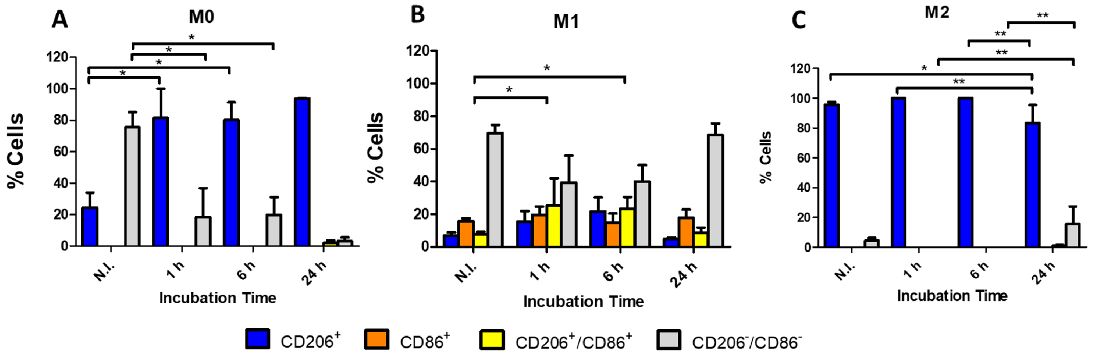

Figure S3. PFCE-NE impact on cell polarization, displayed as percentage of cells positive to various markers, in M0, M1 and M2 cells. The percentage of cells was calculated over the total number of cells in N.I. condition, while on PFCE-NE positive cells only over the incubated ones. Two-Way ANOVA test, Bonferroni post-hoc test (* $p < 0.05$, ** $p < 0.01$, *** $p < 0.001$).

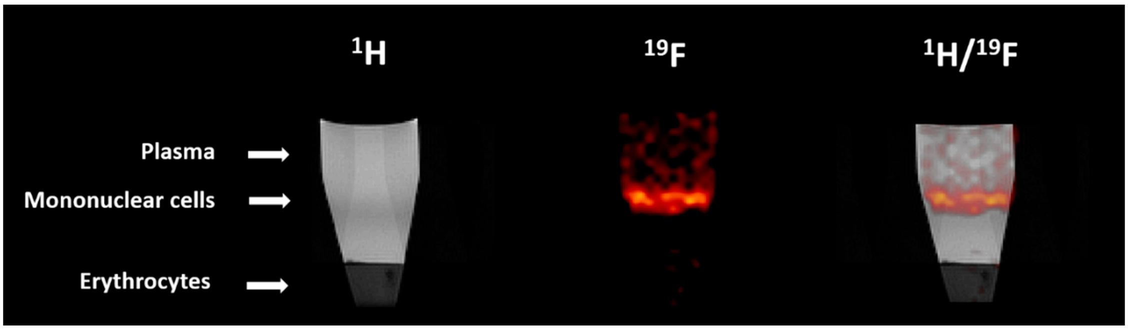

Figure S4. Matching ^{1}H and ^{19}F MR images of a 15-ml Falcon tube after centrifugation of the collected mouse blood over Histopaque density gradient displayed location of ^{19}F signal mainly in mononuclear cells after tail vein injection of 1 mmol/Kg PFCE-NE. Blood samples were collected 3 h after PFCE-NE injection.

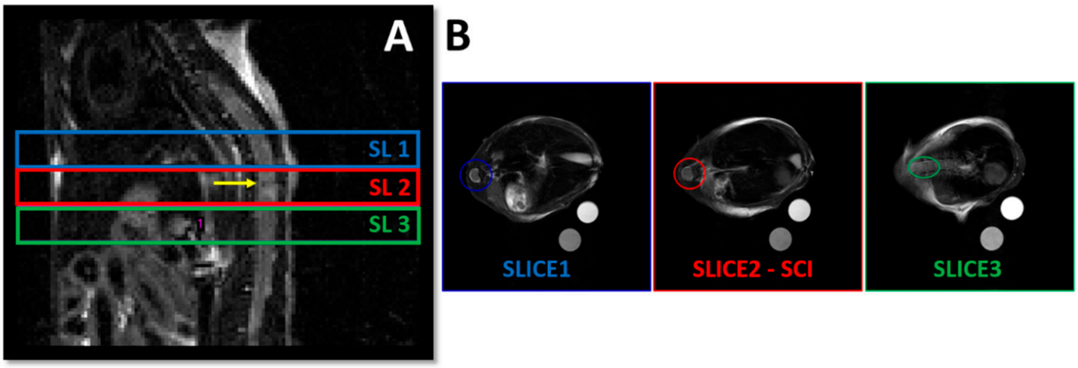

Figure S5. Sagittal (A) and Axial (B) acquisition geometry of ^{1}H/^{19}F MRI. Slice 2 is centered on the lesion (easily located by sagittal ^{1}H MRI –yellow arrow), slice 1 is located above SCI, slice 3 is located below SCI (3.00 mm height per slice). Colored circles indicate spinal cord. White and grey circles correspond to water or PFCE-NE standard reference tube, respectively.

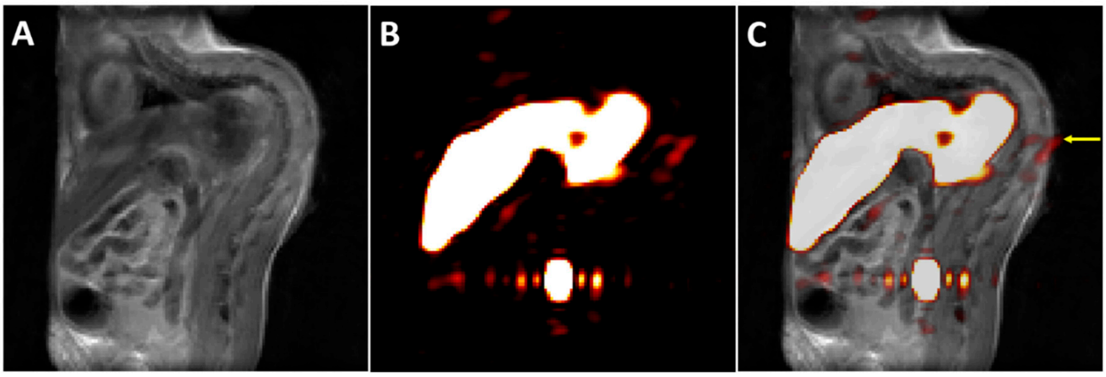

Figure S6. (**A**) ^{1}H, (**B**) ^{19}F, (**C**) ^{1}H and ^{19}F sagittal images of a representative SCI mouse (the lesion is indicated by the yellow arrow). Artifacts coming from the liver and main blood vessels can be noticed in B and C.

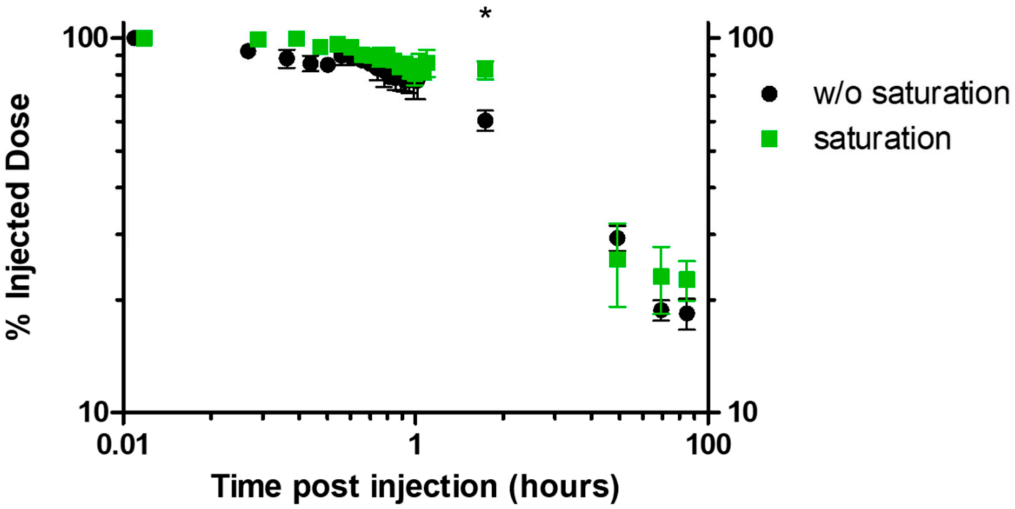

Figure S7. Blood clearance of PFCE-NE with (green squares) or without (black circles) pre-injection of liposomes in order to pre-saturate Kupffer cells, calculated by 19F MRS in healthy mice (n = 6, * $p < 0.05$).

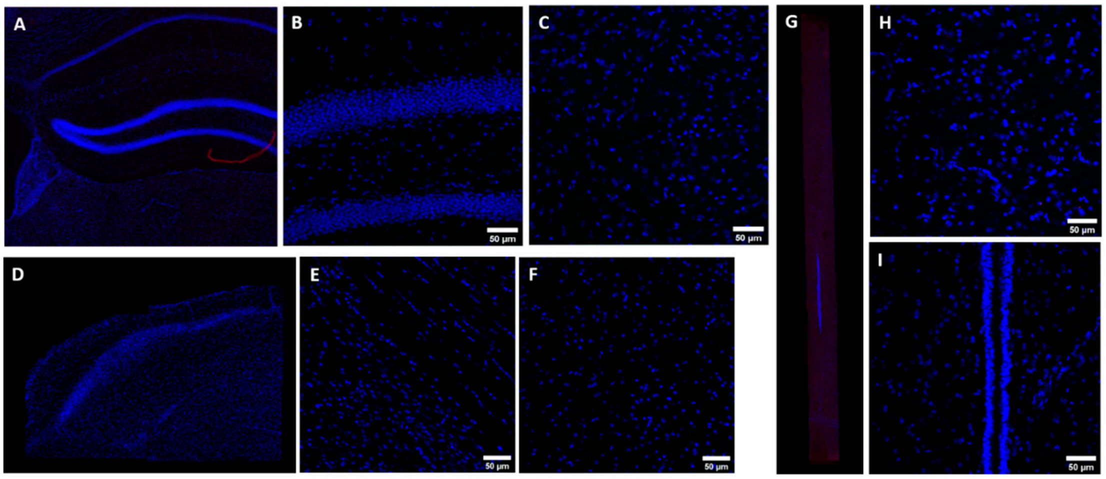

Figure S8. Fluorescence (A, D, G) and confocal microscope (B,C, E,F, H,I) images. (A–C) brain of healthy mice sacrificed 24 h post injection of PFCE-NE; (D–F) brain of healthy mice who received multiple administrations of PFCE-NE and were sacrificed 24 h post the last injection of PFCE-NE; (G–I) spinal cord of healthy mice who received multiple administrations of PFCE-NE and were sacrificed 24 h post the last injection of PFCE-NE. Nuclei are labelled in blue by DAPI. Red channel corresponds to the rhodamine signal of PFCE-NE (not detected).

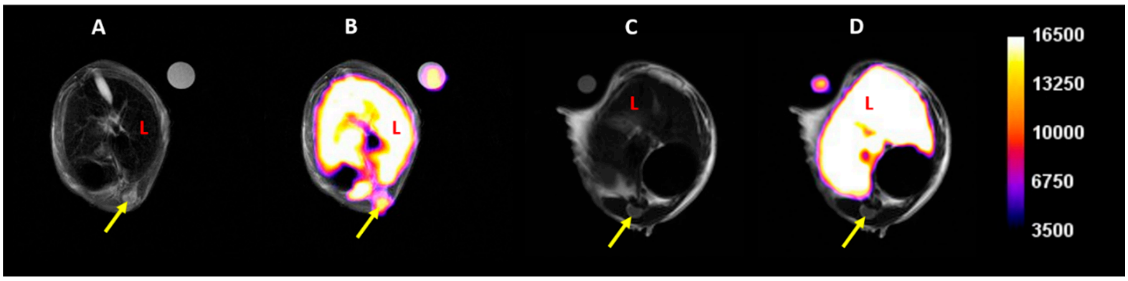

Figure S9. ^{1}H (A, C) and $^{1}H/^{19}F$ (B, D) MRI of SCI (A, B) or healthy (C, D) mouse treated with multiple administrations of PFCE-NE, imaged 24 hours post injection at 14 DPI or 14 days post enrollment, respectively. L corresponds to liver, while the yellow arrows indicate the spinal cord. The gray circles correspond to a standard reference tube containing PFCE-NE.

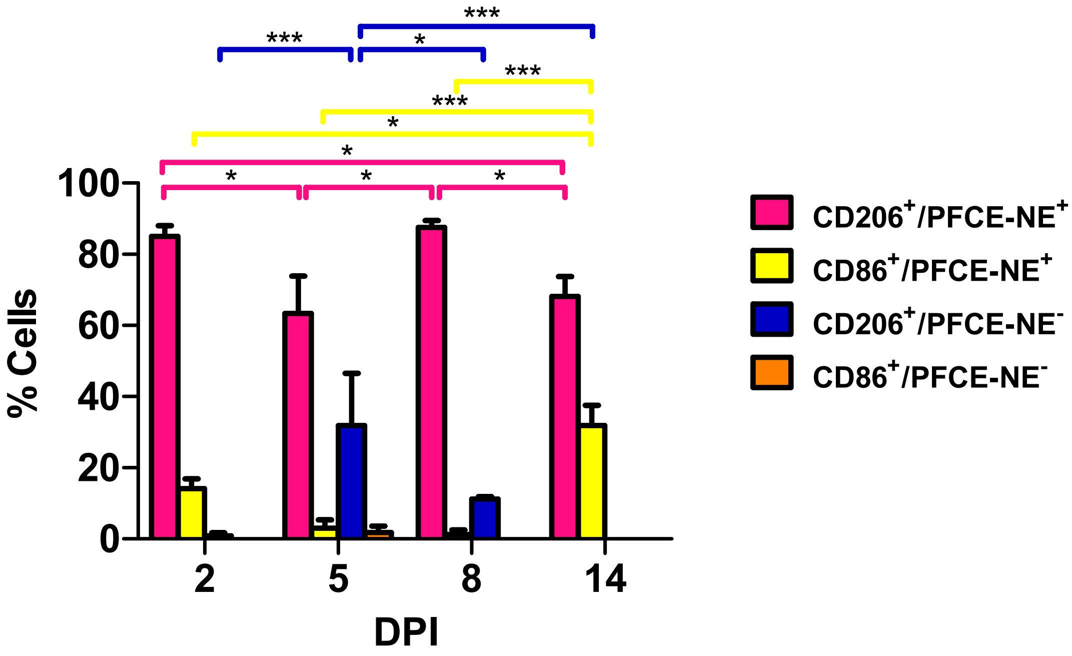

Figure S10. Percentage of M1 (CD86+) and M2 (CD206+) cell populations, either positive or negative for PFCE-NE. Percentages were calculated on the total number of cells. Two-Way ANOVA test, Tukey post-hoc test * $p < 0.05$, *** $p < 0.001$.

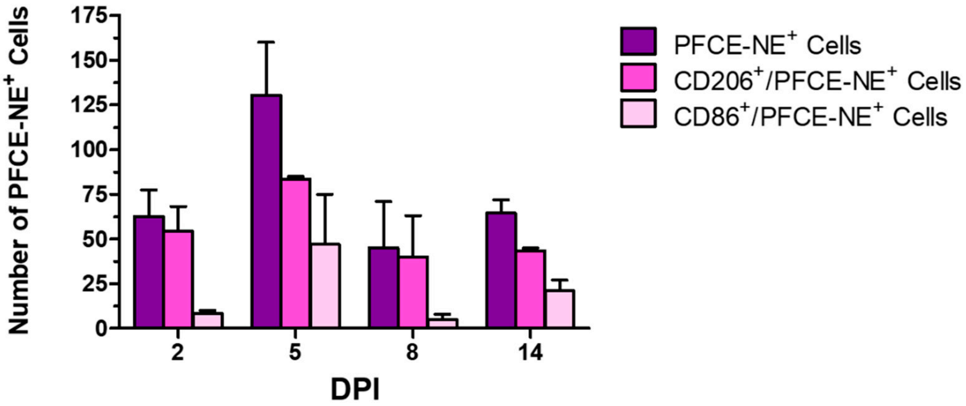

Figure S11. Counts summarizing the average number per frame (analyzed image) of all PFCE-NE+ (purple), CD206+/PFCE-NE+ (violet) and CD86+/PFCE-NE+ (pink) cells located at the injury site at different DPI following single administrations of PFCE-NE. No statistically significant differences are present (One-way ANOVA test, $p > 0.05$).

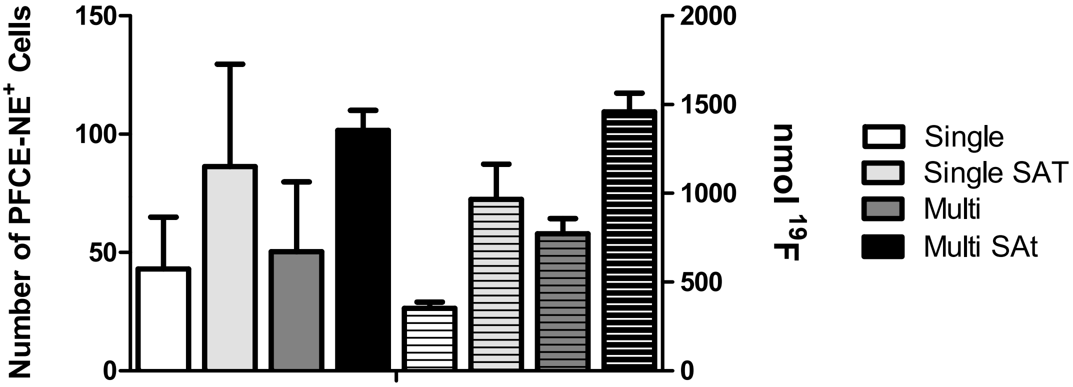

Figure S12. Correlation between the mean number of PFCE-NE+ M1/M2 cells (filled bars) per frame (analyzed image)counted in ex-vivo slices of spinal cords and the number of 19F nmol (striped bars) measured in the injured region by MRI at 14 DPI following different administration protocols.

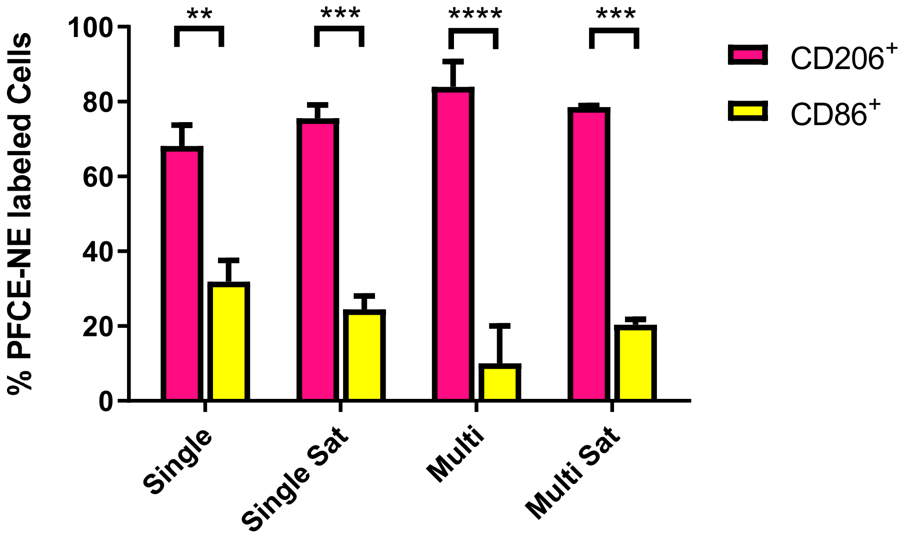

Figure S13. % PFCE-NE labeled cells positive for CD206 (pink bars) or CD86 (yellow bars) counted ex-vivo at 14 dpi in mice treated with different protocols. Two-way ANOVA test, Bonferroni post-hoc test, ** $p < 0.01$, *** $p < 0.001$, **** $p < 0.0001$.
